# Design and Characterization of Peptide-Based Self-Assembling Microgel for Encapsulation of Sesaminol

**DOI:** 10.3390/foods14060971

**Published:** 2025-03-12

**Authors:** Jinhong Gao, Heng Du, Zhenhong Zhang, Qunpeng Duan, Libo Yuan, Bingchao Duan, Hongyan Yang, Kui Lu

**Affiliations:** 1 College of Food Science and Engineering, Henan University of Technology, Zhengzhou 450001, China; jinhonggao@126.com (J.G.); josie_do531@126.com (H.D.); 2 School of Chemistry and Chemical Engineering, Henan University of Technology, Lianhua Road 100, Zhengzhou 450001, China; 13525784069@163.com (Z.Z.); lbyuan@haut.edu.cn (L.Y.); 3 School of Chemistry and Printing-Dyeing Engineering, Henan University of Engineering, Zhengzhou 450007, China; qpduan@haue.edu.cn; 4 School of Food Science and Chemical Engineering, Zhengzhou University of Technology, Zhengzhou 450044, China; duanbc100@163.com (B.D.); hizifu0011@126.com (H.Y.)

**Keywords:** mechanism, interactions, bioaccessibility, antioxidant, molecular docking

## Abstract

Sesaminol is a natural functional compound of sesame with low bioaccessibility due to its high crystallinity. Here, a peptide-based self-assembly microgel was constructed to encapsulate sesaminol, reducing its crystallinity and improving its bioaccessibility. In this contribution, the peptide AcNH-Leu-Tyr-Tyr-CONH_2_ (LYY) was shown to form a mesoporous three-dimensional (3D) microgel through microstructure characterization. Various characterization methods revealed that the LYY peptide self-assembled through *β*-folds and random coils, and the primary intermolecular interactions arose from hydrogen bonding and the π-π stacking effect. Subsequently, sesaminol was encapsulated within the microgel through co-assembly. The maximum encapsulation efficiency of sesaminol was 80.8 ± 0.9%, mainly in the form of nanoparticles encapsulated in microgel by morphology characterization. The XRD results indicated that sesaminol primarily existed in an amorphous state following encapsulation. The cumulative release indicated that sesaminol had a sustained release effect in the encapsulation system. Its bioaccessibility and antioxidant levels were increased. Molecular docking indicated that the main interactions between sesaminol and the self-assembled structure were hydrogen bonding and π-π interactions. Establishing sesaminol encapsulation provides valuable data and theoretical support for the research of sesaminol and the sesame processing industry.

## 1. Introduction

Sesaminol, a lignan predominantly present in sesame oil and glycosylated forms within sesame seeds [1], has recently garnered significant scientific interest owing to its multifaceted biological properties. These include demonstrated antioxidant [2], anti-inflammatory [3], and anti-cancer [4] activities. However, as with many hydrophobic phenolic compounds [5], sesaminol exhibits limited bioaccessibility due to its crystalline structure. Previous research has shown that encapsulation strategies can effectively reduce the crystallinity of hydrophobic bioactive substances and enhance their bioavailability [6,7,8]. Nevertheless, most studies on sesaminol focus on its biological activity, and there are limited reports on developing sesaminol encapsulation systems. Therefore, conducting relevant research on the construction of sesaminol encapsulation systems is crucial.

Although various phenol encapsulation systems have been constructed to solve these shortcomings, there are limited reports on functional encapsulation systems that can stabilize and enhance their biological activity. Peptides, as fundamental biological building blocks, exhibit excellent biocompatibility, controllable degradation, diverse biological activity, and precisely tunable self-assembly behavior, positioning them as superior biomaterial platforms [9,10]. Compared to proteins, peptides demonstrate enhanced synthetic control through their structural simplicity while maintaining comparable biological relevance [11]. Compared to other biomolecules, peptides have excellent biocompatibility and diverse biological activity [9]. These attributes have driven their widespread adoption in drug encapsulation, three-dimensional (3D) cell culture, wound healing dressings, etc. Critically, research indicated that active peptides have a synergistic effect with phenolic compounds [12]. Therefore, peptide-based self-assembled materials were suitable for encapsulating sesaminol [8,13].

Ultra-short peptides have garnered considerable attention in self-assembly research due to their precise adjustability and accessible establishment of structure–property relationships [14]. Various supramolecular structures, such as hydrogel, vesicles, and nanofibers, have been constructed through ultra-short peptide self-assembly [15]. Microgel has the advantages of small size, large specific surface area, easy natural clearance, and enhanced tissue barrier penetration. It can be utilized for transepithelial and local injection and oral and nasal drug delivery [16,17]. However, the preparation of microgel using self-assembled peptides is still not widely carried out due to various difficulties, including its rapid kinetics and infinite self-assembly behavior [18].

Tyrosine (Y) is an essential amino acid for humans and a critical constituent of antioxidant peptides derived from food [19], possessing unique structural features that enable precise control over supramolecular assembly. Its aromatic ring system provides hydrophobicity and π-π stacking interactions—key drivers of peptide self-assembly [20]. Furthermore, the phenolic hydroxyl group facilitates the formation of ordered architectures through directional hydrogen bonding while simultaneously conferring intrinsic antioxidant capacity to the assembled structures [21,22]. This dual functionality positions tyrosine-containing peptides as ideal candidates for enhancing the bioactivity of encapsulated antioxidants like sesaminol. Notably, YY possesses aromatic rings and hydroxyl groups akin to sesaminol, facilitating the interaction between sesaminol and LYY. This is beneficial for enhancing both encapsulation efficiency and bioactive compound stabilization. Previous studies have shown that Fmoc-YY can self-assemble into a hydrogel with excellent antioxidant activity. Fmoc-Y forms a three-dimensional (3D) cocoon-like structure while self-assembling to form a hydrogel [23]. Inspired by this, it is reasonable to speculate that using YY as building blocks, modifying them with various hydrophobic amino acids, and adjusting the self-assembly microenvironment could provide a beneficial structural foundation for obtaining a self-assembled microgel with antioxidant activity.

Selecting amino acids with alkane side chains as hydrophobic tails to modify the hydrophilic heads of peptide chains is a typical strategy for constructing peptide-based assembly materials. However, subtle changes in peptide composition and sequence can cause complex spatial variations in ultra-short peptides by molecular design. Previous studies have shown that using three L to replace isoleucine (I) residues of Ac-I_3_K-NH_2_ results in a self-assembled structure from nanofibers transformed into spherical structures [24]. Other studies have confirmed that the amino acid sequences in amphiphilic tetrapeptides can self-assemble into different 1D structures, including nanoribbons, nanofibers, and nanotubes [25]. Researchers are growing increasingly concerned about the influence of the above factors on the self-assembly behavior of ultra-short peptides. However, the impact of subtle changes in structure on peptide self-assembly behavior from a microscopic viewpoint has not been systematically investigated.

In this work, the self-assembly behavior of a series of YY-derived tripeptides was investigated to construct a self-assembly microgel to encapsulate sesaminol, aiming to effectively encapsulate it, reduce its crystallinity, and enhance its antioxidant and bioaccessibility. Firstly, the LYY microgel was fabricated, and its microstructure was further characterized using transmission electron microscopy (TEM), scanning electron microscopy (SEM), and small-angle X-ray scattering (SAXS). The self-assembly mechanism was elucidated using a fluorescence spectrometer, circular dichroism (CD), nuclear magnetic resonance (NMR) spectra, and molecular dynamics (MDs) simulation. Subsequently, sesaminol was encapsulated in the microgel through co-assembly; its microstructure, encapsulation efficiency, crystallinity, antioxidant activity, release behavior in vitro digestion, bioaccessibility, and the mechanism of its intermolecular interactions were investigated in detail. This research helps provide molecular modules for the construction of peptide-based microgel as a delivery system and to provide scientific support for developing the sesaminol and sesame processing industry.

## 2. Materials and Methods

### 2.1. Materials and Reagents

Amino acids and rink amide resin (loading: 0.76 mmol/g) were purchased from CSBio^®^ (Shanghai, China) Ltd. Deuterated water (D_2_O, D, 99.9%, with 0.05% *V*/*V* TMSP and dimethyl sulfoxide-d6 (DMSO-*d*_6_, D, 99.9%, with 0.03% *V*/*V* TMS) was obtained from Qingdao Tenglong Weibo Technology Co., Ltd. (Qingdao, China). 1,1,1,3,3,3-hexafluoro-2-propanol (HFIP), 8-anilino-1-naphthalenesulfonic acid (ANS), Thioflavin T (ThT), 2,2-Diphenyl-1-picrylhydrazyl (DPPH), and 3-4,5-dimethylthiazol-2-yl)-2,5-diphenyltetrazolium bromide (MTT) were purchased from Aladdin Reagent Co. Ltd. (Shanghai, China). Artificial saliva juice (α-amylase, mucin, uric acid, and so on), artificial gastric juice (pepsin, mucin, and so on), and artificial intestinal fluid (trypsin and 0.1% bile salt from pig) were purchased from Yuanye Bio-Technology Co., Ltd. (Shanghai, China). HEK293 cells were obtained from Procell Life Science & Technology Co., Ltd. (Wuhan, China). All other reagents were analytical grade or higher.

### 2.2. Synthesis and Characterization of Peptides

The peptides Ac-Leu-Tyr-Tyr-NH_2_ (LYY, MW 498.52 g/mol); Ac-Tyr-Leu-Tyr-NH_2_, (YLY, MW 498.52 g/mol); Ac-Tyr-Tyr-Leu-NH_2_ (YYL, MW 498.52 g/mol); Ac-Ala-Tyr-Tyr-NH_2_ (AYY, MW 456.44 g/mol); Ac-Arg-Tyr-Tyr-NH_2_ (RYYN, MW 541.55 g/mol); Ac-Val-Tyr-Tyr-NH_2_ (VYY, MW 484.49 g/mol); Ac-Lys-Tyr-Tyr-NH_2_ (KYY, MW 513.53 g/mol); Ac-Ile-Tyr-Tyr-NH_2_ (IYY, MW 498.51 g/mol); and Ac-Gly-Tyr-Tyr-NH_2_, (GYY, MW 442.47 g/mol) were synthesized by the Fmoc solid-phase synthesis method on rink amide resin according to the previous literature [26]. In addition, acid anhydride was coupled with the N-terminal of the peptide. After all reactions, the peptide was cleaved from the rink resin. The peptides were purified by Yaxian Chemical Co., Ltd. (Shanghai, China). The purified products were detected using semi-preparative liquid chromatography (Agilent 1260, Agilent, CA, USA). The HPLC analysis method is shown in the supporting information. The structure of the peptides was measured by ESI-MS (6456 LC/Q-TOF, Agilent, CA, USA) in positive ion mode and by ^1^H-NMR (400 MHz AVANCE III HD Bruker, Switzerland) (Figures S1–S9). All peptides’ melting points, thermal stability, water solubility, cell viability assay, and antioxidant ability were investigated using the methods outlined in the Supplementary Material.

### 2.3. Preparation of Peptide Microgel

Various YY-derived tripeptides were prepared into a 10 mmol stock solution using 2 mL HFIP as the solvent. Then, 450 μL of the stock solution was transferred into a centrifuge tube, 300 μL of deionized water was added, and the mixture was mixed immediately with a vortex oscillator. The solutions were incubated at 25 °C for 1 h, when peptides were self-assembled into microgels.

### 2.4. Morphology Characteristics

The morphology of the self-assembled peptides was characterized using a TEM (HT7700, Hitachi, Japan) with an accelerated voltage of 100.0 kV, a SEM (Quanta250FEG, FEI, Hillsboro, OR, USA) with an accelerated voltage of 3.0 KV, and an Oxford MFP-3D AFM (Oxfordshire, UK) in tapping mode. The sample solutions’ scattered intensity I(q) was determined using an SAXS system with a 1D Mythen detector (AntonPaarSAXSpace, Graz, Austria) to analyze the pore structure characteristics of the LYY self-assembled microgel.

### 2.5. Fluorescence Spectroscopy Analysis

#### 2.5.1. Intrinsic Fluorescence Assay

A 10 mmol LYY dispersion solution was prepared using HFIP as the solvent. The maximum excitation wavelength (λ_ex_) of LYY was determined using wavelength-intensity 3D scanning mode. The 6 mmol LYY self-assembly solution was prepared according to the description in Section 2.3 and scanned under the maximum λ_ex_. The fluorescence intensity of the 6 mmol LYY dispersion solution was used as the control.

#### 2.5.2. ThT Fluorescence Assay

The ThT stock solution was prepared at a concentration of 50 μmol with deionized water as the solvent. A centrifuge tube mixed 450 μL of LYY stock solution with 300 μL of ThT stock solution. The mixture was incubated at 25 °C for 1 h, and then the spectra were recorded using ThT (20 μmol in water) as the control. Test conditions were as follows: λ_ex_ was 440 nm; the range of λ_em_ was 460–600 nm.

#### 2.5.3. Critical Aggregation Concentration (CAC) Fluorescence Assay

The ANS dye was prepared as a stock solution in HFIP at a concentration of 0.15 mmol. A series of self-assembling solutions of LYY with various concentrations (3.00, 1.50, 0.75, 0.38, 0.19, 0.09, 0.05, 0.02, and 0.00 mmol) were prepared as described in Section 2.3. Then, 10 μL of ANS was added to each sample solution and mixed immediately. The mixtures were measured after incubating for 1 h at 25 °C. Peptide self-assembly solutions without ANS at various concentrations were used as blanks. The fluorescence spectra for testing were as follows: λ_ex_ was 360 nm; the range of λ_em_ was 400–700 nm.

### 2.6. Circular Dichroism (CD) Spectroscopy Analysis

Different concentrations of peptide self-assembly solutions (100 µmol, 150 µmol, and 200 µmol) were prepared following the method described in Section 2.3. The spectra of the samples were determined using a CD instrument (j-1500, JASCO, Tokyo, Japan). All samples were scanned using HFIP aqueous solution (*v*:*v* = 3:2) as the background.

### 2.7. Nuclear Magnetic Resonance (NMR) Spectroscopy Analysis

^1^H NMR spectroscopic analyses and ^1^H-^1^H 2D nuclear Overhauser effect spectroscopy-icy (NOESY) analyses of the samples were performed on a Bruker 400 MHz spectrometer (400 MHz ADVANCE III HD Bruker, Zurich, Switzerland). ^1^H NMR analysis was utilized for structural identification of all peptides using DMSO-*d*_6_ containing 0.03% (*v*:*v*) TMS as an internal standard. NMR titration and 2D NOESY analysis were performed to investigate the self-assembly mechanism of LYY using HFIP and water as solvents. D_2_O containing 0.05% TMSP was placed in an inner liner tube as the internal standard. The ^1^H-^1^H 2D NOESY spectra were obtained via the standard three-pulse sequence, with a mixing time of 600 ms.

### 2.8. Molecular Dynamic (MD) Simulations Analysis

A total of 20 peptide molecules were randomly placed in a 120 Å cube box using PackMOL with a tolerance of 2 Å and HFIP and water (*v*:*v* = 3:2) as filling solvents. Subsequently, Gromacs 5.1.5 was used for MD simulation with the GAFF2 force field to study self-assembly. The simulation system was set in a closed environment of TIP3P water molecules at a temperature of 25 °C, a pH of 4.8, and a pressure of 1.0 bar. All atoms underwent energy minimization using the steepest descent method. A 1000 ps equilibrium simulation was performed using the canonical ensemble (NVT) and the isothermal-isobaric ensemble (NPT). The MD simulation was conducted for 100 ns, with data collected every 2 fs. A linear constraint-solving algorithm limited the covalent bond length to prevent system collapse. The particle-mesh Ewald (PME) method handled long-distance electrostatic interactions [27]. After all simulations were completed, the radius of gyration (Rg), root mean square deviation (RMSD), and solvent access surface area (SASA) were calculated using the gmx.

### 2.9. Encapsulation Efficiency

A 10 mmol peptide stock solution and various sesaminol solutions at different concentrations (1.50, 0.75, 0.38, 0.19, 0.09, 0.05, 0.02, and 0.01 mg/mL) were prepared using HFIP as the solvent. Then, 225 μL of peptide and sesaminol stock solution were mixed. Then, 300 μL water solutions of different pH values (2.0, 3.0, 4.0, 5.0,6.0, 7.0, and 8.0) were immediately mixed. The mixtures were incubated in different temperatures (20, 25, 30, 35, and 40 °C) for different times (5, 15, 30, 60, 120, 240, and 360 min), transferred to 4 mL ultrafiltration tubes (MWCO = 3000 Da), and centrifuged at 15,643× *g* for 20 min. Afterwards, 200 μL of the unfiltered solution of the sample was transferred to a new centrifuge tube, and then 200 μL ethanol was added. According to the previous literature, the mixture was ultrasonicated for 20 min and filtered by a 0.22 μm filter for HPLC detection [28]. Encapsulation efficiency was calculated using the following Equation (1).

Encapsulation efficiency (%) = (C_0_ − C_1_) × 100/C_0_ (1)

where C_0_ represents the initial concentration of sesaminol in a mixture solution and C_1_ represents the concentration of sesaminol in the permeate solution.

### 2.10. Crystallinity Analysis

The crystallinity of dried sesaminol, LYY, sesaminol–LYY composite microgel, and the physical mixtures of sesaminol and LYY were analyzed and evaluated by XRD analysis (XRD Smart Lab SE, Rigaku, Tokyo, Japan). The scanning range of angles (2θ) was 5° to 40°, and the scanning rate was 10°/min.

### 2.11. Antioxidant Ability

The sesaminol–LYY microgel was prepared and dried at 25 °C. The sesaminol and sesaminol–LYY microgel powder were dissolved in ethanol to evaluate their antioxidant ability; the details of the method are recorded in the Supplementary Material.

### 2.12. Simulated In Vitro Digestion

The simulations were performed according to the methods detailed in prior literature with minor modifications [29,30]. Briefly, the sesaminol–LYY co-assembly microgel was prepared and dried at room temperature under nitrogen. Then, 10 mg of sesaminol–LYY co-assembly microgel powder and sesaminol–LYY physical mixed powder were dispersed in 1 mL of artificial saliva and shaken for 2 min at 37 °C and 100 r/min. Then, 2 mL of artificial gastric juice (pH 1.3) was added and shaken for 2.0 h (37 °C, 100 r/min). Following gastric digestion, 3 mL of artificial intestinal fluid (pH 7.0) was added to the digestion system and shaken for 2.0 h at 37 °C and 100 r/min. During this period, 0.5 mL of release medium was extracted every 20 min, and an equal volume of fresh simulated liquid was introduced. Finally, the release medium was dried in a vacuum freeze dryer for 24 h. Subsequently, 0.5 mL of methanol solution was added, followed by ultrasonic-assisted dissolution. Following filtration through a 0.22 μm organic filter, HPLC was used for detection and analysis following previous reports [28]. The cumulative release ratio of sesaminol was calculated using Equation (2).

Cumulative release ratio = m_t_/m × 100% (2)

where m represents the total mass of sesaminol in the microgel and m_t_ represents the mass of sesaminol released in the simulated liquid.

### 2.13. Bioaccessibility Analysis

The digestion solution was centrifuged for 1.5 h at 4000 rpm and 4 °C to obtain the bioaccessible fraction [31]. The concentration of sesaminol was determined by HPLC [28]. The bioaccessibility of sesaminol was determined according to the following Equation (3).

Bioaccessibility (%) = (Sesaminol in bioaccessible)/(Total sesaminol in microgel)(3)

### 2.14. Molecular Docking

The individual LYY and the stable LYY aggregates obtained through MD simulations, as outlined in Section 2.8, underwent molecular docking analysis using AutoDock4.2 software to establish docking models between individual LYY and sesaminol, as well as between LYY aggregates and sesaminol structures. The box size was set to a cube with a side length of 45 Å and a step size 0.375. The coordinates of the docking box were center_x = −2.14, center_y = 5.45, and center_z = −9.02. The maximum limit for searching conformations was 10,000. A genetic algorithm was employed for conformational sampling and scoring, with the optimal conformation selected based on docking scores.

### 2.15. Statistical Analysis

Each experiment was measured three times, and the results were expressed as mean ± standard deviation (SD). IBM SPSS software (version 19.0, SPSS Inc., Chicago, IL, USA) was used for statistical analysis. The one-way ANOVA and Duncan test were employed to examine significance. Different letters represented a significant difference between the groups (*p* < 0.05).

## 3. Results and Discussion

### 3.1. Sequence Design and Characterization of Peptides

YY with antioxidant activity and a particular structure was selected as the core sequence to modify to prepare a functional peptide-based self-assembly structure. The rigid structure of aromatic rings in Y can provide hydrophobicity and π-π stacking for the self-assembly of peptides [20]. The hydroxyl group of Y can facilitate peptide assembly into more ordered structures by making hydrogen bonds. Moreover, YY has been widely used to fabricate versatile bio-inspired materials [32]. The lack of flexibility in YY’s structure weakened its self-assembly ability. Therefore, we selected amino acids with flexible alkane side chains to modify YY to obtain functional self-assembled 3D structures.

A series of YY-based derived tripeptides were designed and synthesized based on the above considerations, including LYY, YLY, YYL, AYY, RYY, VYY, KYY, IYY, and GYY. The structures of the peptides were identified using ^1^H-NMR and ESI-MS. The purity of the peptides was greater than 95% (Figures S1–S9). The peptides exhibited high thermal stability (Table S1). The water solubility of the peptides was closely related to their self-assembly ability. The results indicated their water solubility: VYY < IYY < YLY < LYY < YYL < AYY < GYY < RYY < KYY. Moreover, the peptides exhibited good biocompatibility through cell viability tests in HEK-293. LYY and YYL exhibited better antioxidant abilities than the other peptides (Table 1). This may be attributed to their structure being more conducive to interactions with DPPH free radicals. The above results suggested that the peptides had great potential for constructing encapsulation systems for sesaminol.

### 3.2. Self-Assembly Behavior of Peptides

#### 3.2.1. Effect of YY Modified by Amino Acids with Different Length Alkane Chains

Hydrophobic alkane side chains are essential in self-assembling peptides [33]. Therefore, amino acids with hydrophobic alkane side chains of varying lengths (L, I, V, A, and G) were specifically chosen for modifying YY (Figure S10). TEM images indicated that the YY-derived tripeptides with an alkane side chain containing only one methyl and H (AYY and GYY) only form small fiber structures (Figure S10e,f). With the extension of the alkane side chain, which had two methyl groups (VYY), the tripeptides can self-assemble to form 1D fibers (Figure S10d). The further extension of the alkane chain (LYY and IYY) resulted in forming a sea urchin-like 3D self-assembled structure (Figure S10b,c). These results indicated that the alkane chain length was critical to self-assembly. Longer hydrophobic alkyl chains are more effective at promoting the self-assembly of YY-derived peptides because of the enhanced hydrophobic interactions and flexibility of the alkane side chains [14].

To further investigate the impact of hydrophobic groups at the ends of alkane chains on the self-assembly of peptides, the tail of YY was modified with the amino acids K and R. These amino acids possessed alkane side chains but exhibited hydrophilic amino and guanidine groups at the termini of their alkane chains, respectively. It was determined that the YY-derived tripeptides only self-assembled into small structures (Figure S10g,h). This finding highlights the crucial role of hydrophobic groups at amino acid terminals in self-assembly by providing substantial hydrophobic forces.

The impact of L’s specific position in the peptide sequence on the self-assembly behavior of YY-derived tripeptides was examined. It was found that the YY-derived tripeptide could form a sheet or small self-assembly structure when L was in the middle (Figure S10i). The tripeptide could form a 1D filamentous disordered structure when L was at the head (Figure S10j). This result suggested that the position of the alkane chain was critical to the self-assembly structure.

In summary, the TEM results showed that LYY and IYY formed a distinct self-assembly structure. LYY exhibits superior antioxidant activity compared to IYY, prompting further research into its self-assembly.

#### 3.2.2. Micromorphology Characteristics of LYY Self-Assembly Structure

**Figure 1:**
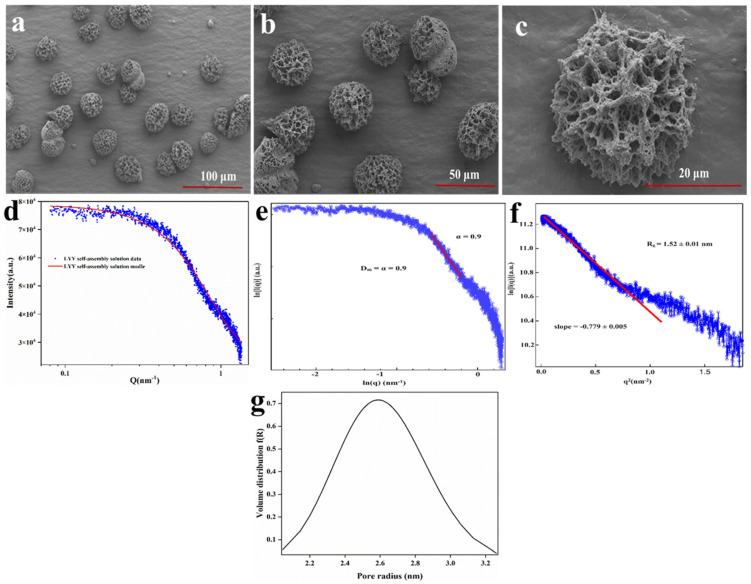
SEM images of the LYY self-assembly microgels, magnification (**a**) 1.0 k×, (**b**) 2.0 k×, (**c**) 10.0 k×. SAXS spectra of the LYY microgel: (**d**) fractal characteristics of the LYY microgel, (**e**) the Guinier curve of the LYY microgel, (**f**) the void size of the LYY self-assembly structure, (**g**) pore radius distribution of the LYY microgel.

To further investigate the micromorphology of the LYY self-assembly structure, SEM was employed to observe the 3D microstructure at an HFIP to H_2_O ratio of 3:2. The results indicated that LYY self-assembled into micron-sized spherical 3D structures, with uniform pore size and distribution (Figure 1a–c). Moreover, the nanofibers wind together to form the 3D structure. The self-assembly process consisted of peptide-based hydrogels [34]. The micromorphology of the peptide-based self-assembly particles corresponds to the microgels described in the previous literature [35].

SAXS was employed to further investigate the detailed characteristics of the pore structure in the LYY self-assembled microgel. The small-angle diffraction spectra of the peptide self-assembled microgel are presented in Figure 1d. The fractal characteristics of LYY self-assembly and the slope in the low-q region were obtained through linear fitting using the least squares method (Figure 1e). The slope value of the sample was 0.9, which conformed to the pore fractal characteristics (0 < α (0.9) < 3) [36]. This also implied that the LYY self-assembly structure was loose and porous. The void size of the LYY self-assembly structure was determined to be 1.52 ± 0.01 nm (Figure 1f). The results indicated that the pores exhibited a spherical morphology and an approximately normal distribution. The average pore diameter was 2.6 nm, suggesting the microgel possessed a mesoporous structure (Figure 1g) [28].

The mesoporous structure of LYY self-assembled microgels resulted in a high specific surface area, thus effectively providing more interaction sites [37]. These characteristics suggest that the supramolecular self-assembly microgel has significant potential for applications in nutrient and drug encapsulation, sustained release, and beyond. Therefore, further research into the self-assembly mechanism could prove valuable.

### 3.3. Mapping the Mechanism of LYY Microgel

#### 3.3.1. Fluorescence Spectroscopy

**Figure 2:**
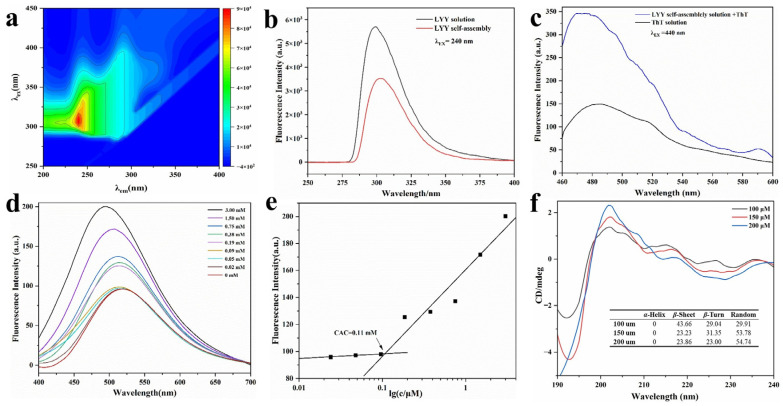
Fluorescence and CD spectra of LYY self-assembles. (**a**) Complete scans of λ_ex_ and λ_em_. (**b**) Fluorescence emission spectra of LYY HFIP–water binary solution and LYY dispersion. (**c**) Fluorescence emission spectra of ThT–LYY solution and ThT solution. (**d**) Fluorescence emission spectra of ANS-LYY at various concentrations (0–3 mmol). (**e**) CACs of LYY; (**f**) CD spectra and secondary structure ratio of LYY at various concentrations (100, 150, and 200 μmol).

The fluorescence signal of the LYY solution was determined. The maximum *λ*_ex_ of LYY was 240 nm (Figure 2a). It was found that with the same concentration of peptide solution, the fluorescence signal intensity in the self-assembled system decreased significantly compared to the peptide solution (Figure 2b). The fluorescence-quenching phenomenon was primarily attributed to π-π stacking of benzene rings among peptide molecules, indicating that such stacking might occur during the self-assembly process of LYY [26,38].

A ThT assay was performed using fluorescence spectroscopy (Figure 2c). The ThT solution showed weak fluorescence absorption at *λ*_ex_ 440 nm and *λ*_em_ 485 nm. The fluorescence intensity significantly increased when ThT was combined with LYY self-assembly. This finding indicated the presence of *β*-sheets in the LYY self-assembly microgel [39].

The fluorescence spectra of peptide solutions with varying concentrations co-incubated with ANS were analyzed to determine the CAC of peptide self-assembly. The fluorescence signal of 1,8-ANS displayed a blue shift as the peptide concentration rose, particularly with a marked increase between 0.09 and 0.19 mmol (Figure 2d). The results indicated that the CAC range of the peptides was 0.09–0.19 mmol. Hydrophobic forces were involved, and hydrophobic groups formed hydrophobic cavities during self-assembly [40]. The peak fluorescence intensity at each concentration was recorded as the Y value, while the logarithmic value of each peptide concentration was recorded as the X value. A linear fitting analysis was subsequently performed. The CAC of peptide-forming aggregates was 0.11 mmol, according to the results of the linear fitting analysis (Figure 2e).

#### 3.3.2. Circular Dichroism (CD) Spectroscopy

The spectra of varying peptide concentrations (Figure 2f) exhibited a minimum at approximately 195 nm, indicating random coils, and a significant peak at 200 nm, suggestive of *β*-strand conformation [41]. The SELCON 3 in CDPro software was employed to further calculate the secondary structure content of self-assembled peptides (Figure 2f). The results indicated that the peptide self-assembly exhibited three main secondary structures: *β*-sheets, *β*-turns, and random coils. When the concentration of 100 μmol was lower than the CAC of LYY, the main secondary structure of the self-assembly was the *β*-sheet. With the peptide concentration increasing to 200 μmol, the percentage of *β*-sheet decreased to 23.86%, while the random coils increased to 54.74%. In addition, the proportion of different secondary structures tended to stabilize with increasing concentration. The findings showed that *β*-sheets and random coils are crucial for the self-assembly of peptides [42]. These results confirmed the findings of the ThT assay. Moreover, the random coils were beneficial to promoting the formation of spherical micelles [43].

#### 3.3.3. Nuclear Magnetic Resonance (NMR) Spectroscopy

**Figure 3:**
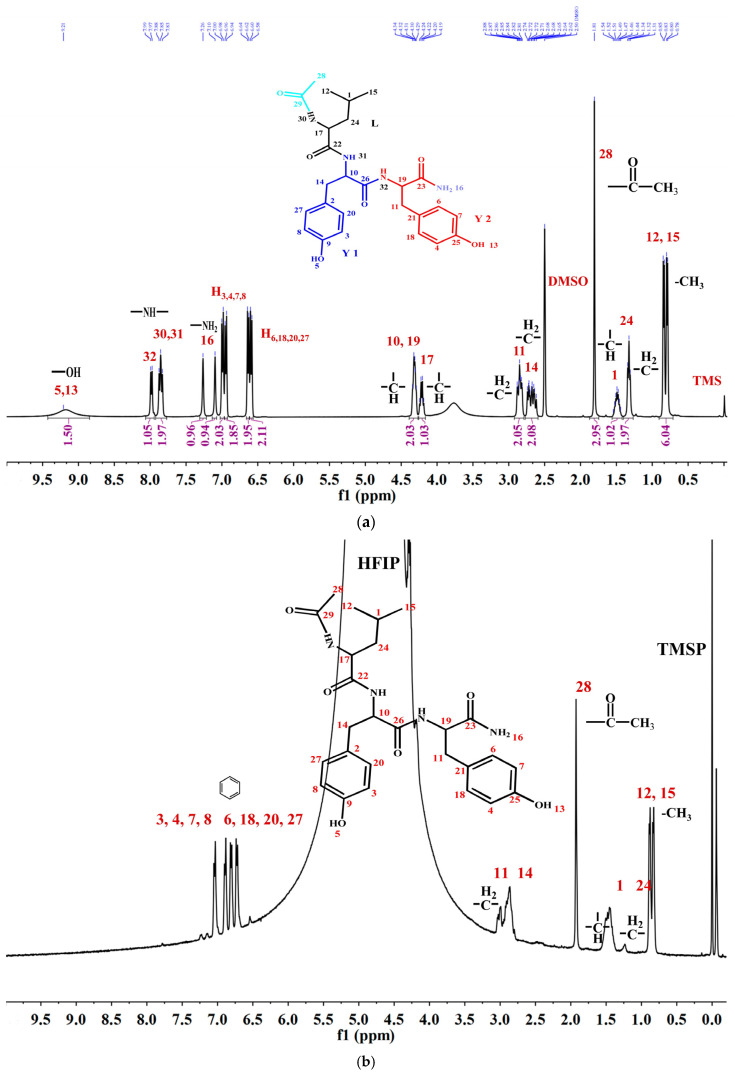

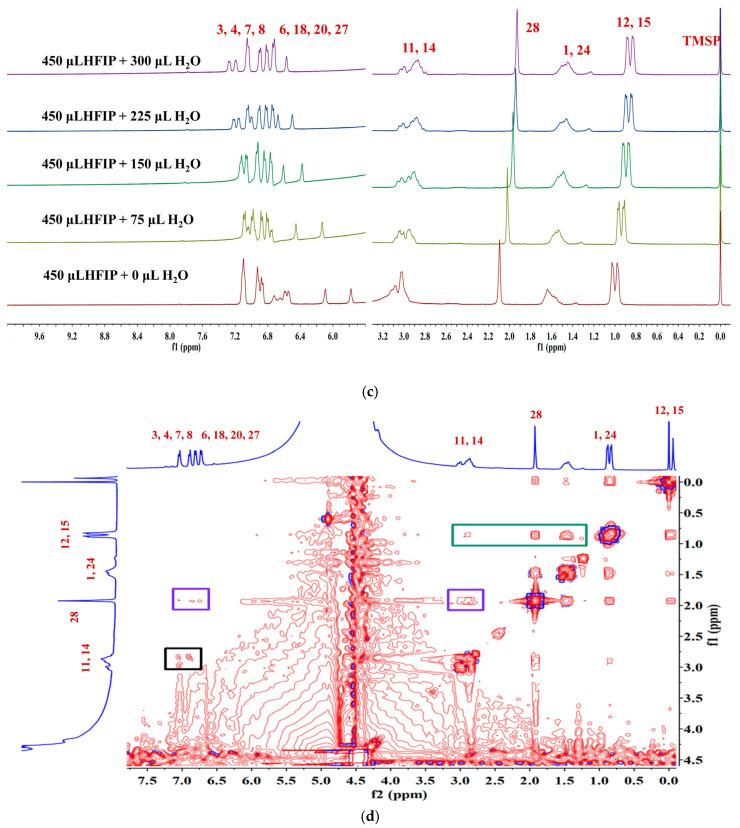
NMR spectra of the LYY solution. (**a**) ^1^H NMR spectra of LYY with DMSO-*d*_6_ as the solvent. (**b**) ^1^H NMR spectra of LYY with HFIP as the solvent. (**c**) ^1^H NMR titrations of the LYY–HFIP solution with water (volume of water: 0, 75, 150, 225, 300 μL). (**d**) ^1^H-^1^H 2D NOESY spectra of LYY under solvent ratio HFIP:water = 3:2 (6 mmol) at 25 °C.

To thoroughly investigate the self-assembly mechanism of LYY, the ^1^H NMR spectrum of LYY (DMSO-*d*_6_ as the solvent) was first measured to identify the chemical shift of each hydrogen proton on the peptide chain. The carbon atoms were labeled with numbers (Figure 3a). The ^1^H NMR titrations were carried out by gradually adding water (0, 75, 150, 225, and 300 μL) to the LYY_–_HFIP solution to investigate the influence of the solvent on H-bond formation during the dynamic self-assembly of peptides (Figure 3b,c) [44]. Compared to the solvent HFIP without water, the peak positions of hydrogen protons corresponding to the aliphatic (H_3,4,6,7,8,18,20,27_) and aromatic (H_3,4,6,7,8,18,20,27_) groups of LYY gradually shifted towards lower fields with increasing water addition, indicating that the additional water could weaken or disrupt the H-bonds between HFIP and LYY [45].

^1^H-^1^H 2D NOESY NMR is crucial for investigating intermolecular interactions during self-assembly [46]. In general, the appearance of the NOE signal showed that the distance between H protons was less than 5 Å [47]. The ^1^H-^1^H 2D NOESY spectra of LYY (HFIP: water = 3:2) are shown in Figure 3d. The results showed cross signals between H_12,15_ and H_1,24,28,11,14_, denoted with a green box, indicating that the two methyl groups of the flexible alkane chain of Leu were closed to the methylene and methine groups of the Leu side chain, the methyl groups obtained after anhydride termination, and the methylene groups of Y1 and Y2. The cross signals between H_28_ and H_3,4,6,7,8,20,27_ and H_11,14_ indicated that methyl was near the rigid benzene rings of Y1 and Y2, marked with purple boxes. The short distance between H_11,14_ and H_3,4,6,7,8,20,27_, marked with a black box, indicated possible π-π stacking interactions among the rigid aromatic groups. In addition, the signal peaks of H_10,17,19_ on the peptide chain are covered by the signal peaks of hydrogen protons from HFIP. Thus, whether these hydrogen protons have interacted with other H protons in the peptides could not be determined. In conclusion, the results indicated that the three methyl groups (H_12,15,28_) at the N-terminus were essential for self-assembly. These groups are more likely to cause flexible hydrocarbon chains between molecules to fold or wind, bringing rigid aromatic rings closer together, which then aggregate further to form a 3D network structure microgel.

#### 3.3.4. Molecular Dynamic (MD) Simulations

**Figure 4:**
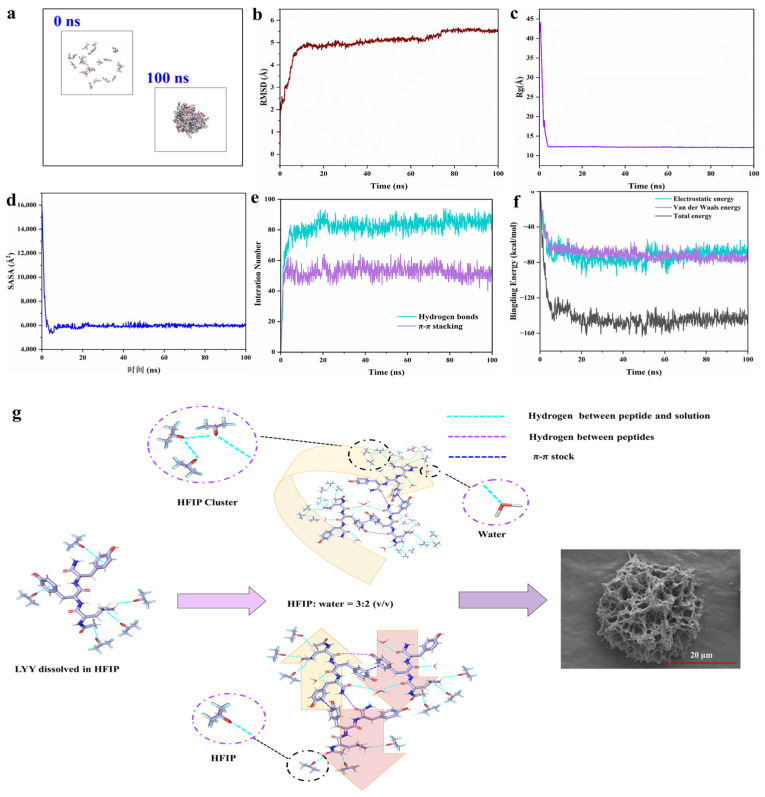
MD simulation of the peptide self-assembly process. (**a**) Structural change images (0 and 100 ns). (**b**) Time evolution of RMSD. (**c**) Time evolution of Rg. (**d**) Time evolution of SASA. (**e**) Interaction number (hydrogen bonds and π-π stacking). (**f**) Time evolution of the non-bonded energies, including electrostatic interactions (Coulomb), van der Waals interactions (vdW), and total binding energy. (**g**) Schematic diagram of the molecular arrangement of LYY self-assembly.

To further investigate the self-assembly mechanism of LYY at the molecular level, MD simulations of the peptide self-assembly process were performed within 100 ns (Figure 4a and Figure S12a) [27]. The RMSD results showed that self-assembly achieved stability with values consistently remaining within the range of 5–6 Å (Figure 4b). The Rg value decreased significantly from 41.95 Å at 0 ns to 12.08 Å at 100 ns (Figure 4c). SASA exhibited a significant decreasing trend, like Rg (Figure 4d). These results confirmed that the gaps between molecules were significantly reduced during the self-assembly process of peptide molecules. The area of the self-assembled body exposed to solvents was diminished [48,49]. According to the above results, the self-assembly of LYY reached an equilibrium state at 100 ns [50].

MD simulations revealed the formation of hydrogen bonds and π-π stacking interactions between molecules (Figure 4e). The results were consistent with the findings from the NMR and endogenous fluorescence assays. The more significant number of hydrogen bonds, in contrast to π-π stacking, indicated that hydrogen bonds were the primary driving force behind self-assembly [51]. Specifically, the average number of H-bonds and π-π stacking were 85.1 and 50.9, respectively, in the 90–100 ns range. The carbonyl group in the molecule could act as a hydrogen bond acceptor. The hydroxyl group in phenol and the amino group in the peptide chain may serve as hydrogen bond donors (Figure S12b). In addition, the benzene ring of Y could use π-π stacking to promote peptide molecules’ self-assembly and aggregation. The aromatic rings of phenols in the molecule could form π-π stacking interactions (Figure S12c). Gmx_energy was used to investigate the changes in binding energy during self-assembly [52]. The results indicated that the self-assembly process was mainly driven by electrostatic (Coulomb) and van der Waals forces (Figure 4f), with average values of −68.3 kcal/mol and −75.1 kcal/mol, respectively, for 90–100 ns.

According to the results presented above, the schematic illustration of the self-assembling behavior of the LYY in the HFIP–water binary solution is shown in Figure 4g. LYY molecules were closely arranged in a parallel or antiparallel manner due to intermolecular π-π stacking and hydrogen bonds. When solvent HFIP was mixed with water, HFIP formed molecular clusters. The alkane side chains coupling with acid anhydrides displayed strong hydrophobic interactions and flexibility. The hydrophobic groups interacted with the trifluoromethyl (CF_3_) groups through the CH···FC hydrogen bonds. This molecular cluster promoted random coils of peptide and further formed self-assembly into a 3D mesoporous network structure.

### 3.4. Encapsulation Efficiency of Sesaminol in LYY-Microgel

**Figure 5:**
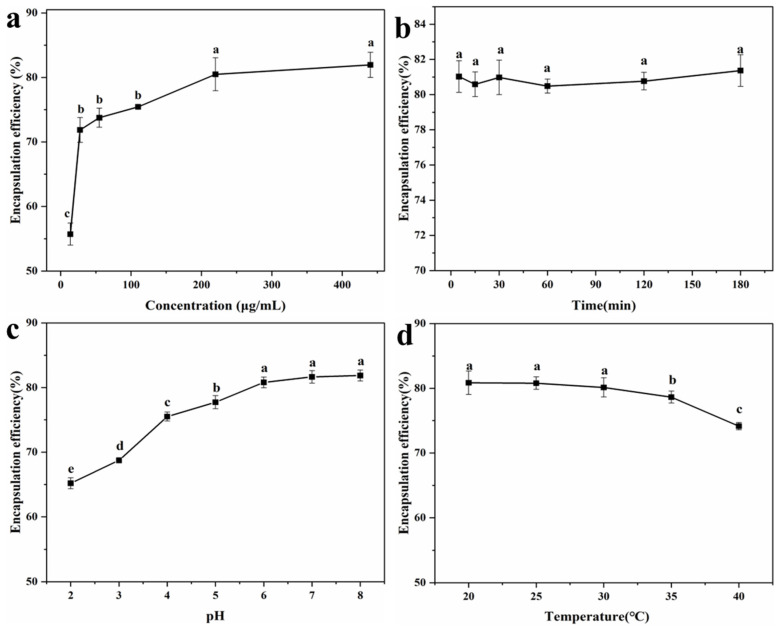
Encapsulation efficiency of sesaminol (**a**) at different concentrations; (**b**) at different action times; (**c**) at different pH; (**d**) at different temperatures (significant differences are denoted as *p* ≤ 0.05 and non-significant differences as *p* > 0.05). a–e: Different letters represented a significant difference between the groups (*p* < 0.05).

To explore the encapsulation effect of LYY-microgel on sesaminol, the impact of sample concentration, contact time, pH, and temperature on the encapsulation efficiency of sesaminol was investigated (Figure 5). As the sample concentration increased from 13.8 μg/mL to 220.1 μg/mL, the encapsulation efficiency of sesaminol improved significantly. The highest encapsulation efficiency was 80.5 ± 2.6% at the sesaminol concentration of 220.1 μg/mL (Figure 5a). As the concentration increased further, the encapsulation efficiency tended to stabilize [53]. Contact time (5–180 min) did not significantly affect the efficiency of encapsulating sesaminol, indicating that the peptide-based microgel rapidly encapsulated sesaminol (Figure 5b). This may be attributed to non-covalent molecular interactions between sesaminol and peptide, such as hydrogen bonds, hydrophobic interactions, and π-π stacking [54,55]. The encapsulation efficiency of sesaminol significantly increased as the pH increased from 2.0 to 6.0 (Figure 5c). As the pH continued to improve, there was no significant change in the encapsulation efficiency of sesaminol. The results indicate that assembling encapsulation systems around pH 7.0 is more conducive to interacting with peptide molecules and solvents [56]. Temperature had no significant effect on the encapsulation efficiency of sesaminol within the range of 20–30 °C (Figure 5d). When the temperature was beyond 35 °C, the encapsulation efficiency decreased. This is related to the fact that low temperature is more conducive to the gel of peptides [42]. The encapsulation conditions were determined by the single-factor test and actual experimental operation: the concentration of sesaminol was 220.1 μg/mL, the contact time was 1 h, the pH was 6.0, and the temperature was 25 °C. Under this condition, the optimal encapsulation efficiency of 80.8 ± 0.9% is superior to the 50.1% of anthocyanin encapsulated by starch-microgel and the 43.8% of lutein encapsulated by self-assembled albumin nanoparticles reported in the previous literature [5,57]. The results indicated that the LYY-based microgel effectively encapsulates sesaminol.

### 3.5. Morphology Characteristics of Sesaminol–LYY Microgel

**Figure 6:**
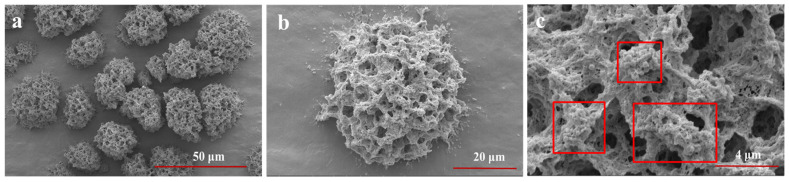
The SEM images of sesaminol–LYY microgel at different magnifications. (**a**) 3.0 k×, (**b**) 10.0 k×, (**c**) 40.0 k×.

SEM characterized the sesaminol–LYY microgel, and the results showed that sesaminol was closely combined with the microgel fiber in the form of nanoparticles (Figure 6), indicating that sesaminol may primarily bind to LYY through interactions during the co-assembly process. Previous reports have demonstrated that hydrophobic compounds in the form of nanoparticles with low crystallinity are beneficial for improving their bioaccessibility, thereby enhancing their bioavailability [58]. Furthermore, the fiber structure of the LYY-based microgel was not destroyed after co-assembly with sesaminol. Compared to before encapsulation, the morphology of the LYY-based microgel changed slightly, consistent with the conformational adaptability of soft materials [59]. This change may result from the interaction between sesaminol and LYY during encapsulation.

### 3.6. Crystallinity

**Figure 7:**
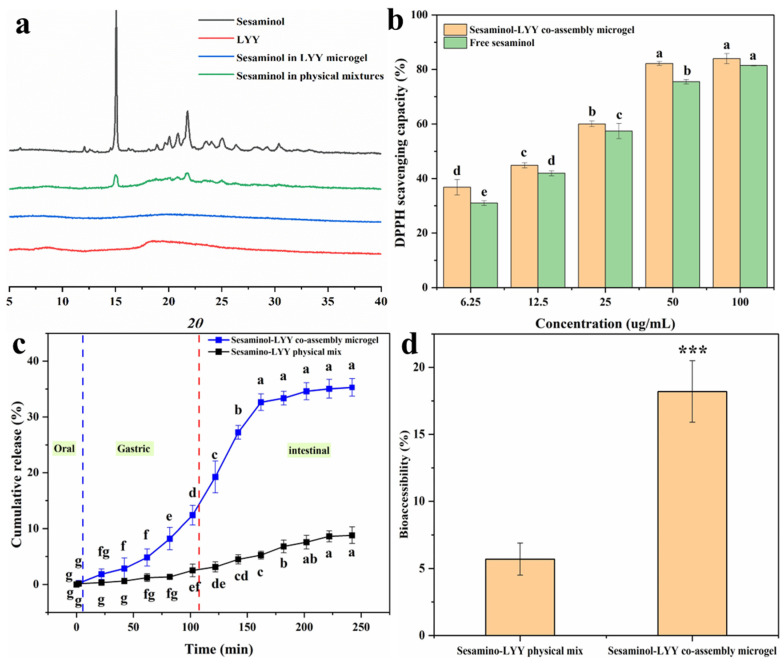
XRD patterns of sesaminol, LYY, sesaminol–LYY physical mixed and sesaminol–LYY co-assembly (**a**). Antioxidant activity of sesaminol and sesaminol–LYY co-assembly microgel (**b**). In vitro release of sesaminol in sesaminol–LYY physical mixed and sesaminol–LYY co-assembly microgel (**c**). Bioaccessibility of sesaminol in sesaminol–LYY physical mixed and sesaminol–LYY co-assembly microgel (***: *p* < 0.001) (**d**). a–g: Different letters represented a significant difference between the groups (*p* < 0.05).

Figure 7a presents the XRD spectra of sesaminol, LYY, the sesaminol–LYY composite microgel, and the sesaminol–LYY physical mixtures. Sesaminol exhibited significant diffraction peaks in the 10–35 nm range, indicating a structure of highly crystalline nature [60]. The diffraction pattern of LYY displays two broad peaks at approximately 8.6 nm and 18.4 nm, characteristic of amorphous compounds. The diffraction pattern of physical mixtures of LYY and sesaminol showed a decrease in peak intensity. Nevertheless, the characteristic peaks of the two samples remain obvious, and the clear overlap of the spectra suggests that complexation has not taken place. In the spectrum of the sesaminol–LYY co-assembly microgel, the characteristic peak of sesaminol has entirely disappeared, indicating that the crystal structure of sesaminol has been disrupted and that the sesaminol in the microgel has transformed into an amorphous state [61]. The sesaminol–LYY co-assembly microgel exhibited halo characteristics, signifying increased amorphousness. This suggested that the molecular structure of LYY and sesaminol changed during the assembly process [61].

### 3.7. Antioxidant Activity

The DPPH scavenging rate of samples was investigated to evaluate their antioxidant activity (Figure 7b). The samples displayed excellent free radical scavenging ability. When the concentration of sesaminol was 100 µg/mL, both samples’ free radical scavenging rates exceeded 80%. Their antioxidant capacity was significantly enhanced with increasing concentration. Furthermore, the antioxidant activity of the sesaminol–LYY co-assembled microgel was superior to that of free sesaminol. This can be attributed to the antioxidant capacity of LYY microgel (Figure S11). However, the increase in antioxidant capacity was limited. This may be attributed to the strong interaction between sesaminol and LYY, arising from hydrogen bonding or π-π stacking [54].

### 3.8. In Vitro Simulated Digestion

In vitro simulated digestion experiments were performed to investigate the release behavior of sesaminol during digestion in both the physical mixture of sesaminol–LYY and the co-assembly of sesaminol–LYY (Figure 7c). HPLC chromatograms of simulated digestion are shown in Figure S13. During the oral phase, the release rate of sesaminol from the co-assembled microgel was low, primarily due to the brief contact time of the sample in artificial saliva and the minimal enzymatic effect of amylase on the peptide-based microgel. During the digestion stage of the simulation, the cumulative release of sesaminol from the co-assembled microgel reached 12.4%. Partial sesaminol was released due to the dissolution of the peptide-based microgel under the combined action of acid and pepsin. However, the hydrophobic cavity and steric hindrance in the microgel impeded the interaction with the protease, resulting in a limited release amount [61]. During the intestinal digestion stage, the cumulative release rate of sesaminol significantly increased to 35.29% and tended to stabilize. The rapid release of sesaminol indicated that the peptide-based microgel disintegrates rapidly under the influence of trypsin [62]. However, the microgel’s network structure and hydrophobicity facilitated controlled release during the intestinal phase. Compared to the physical mixture of sesaminol–LYY, the cumulative release rate at the end of digestion increased 2 times. This is mainly attributed to the nanoparticles and amorphous form of sesaminol in the encapsulation system [31].

### 3.9. Bioaccessibility

Bioaccessibility is crucial for evaluating substance efficacy, especially in nutrition and pharmaceutical research [63]. Therefore, the effect of LYY microgel on the bioaccessibility of sesaminol was assessed through simulating in vitro digestion (Figure 7d). The accessibility of sesaminol in physical mixtures was merely 5.7%, mainly due to its high crystallinity. However, the bioaccessibility of sesaminol rose to 18.2% when encapsulated in microgel, which is 3.19 times that of the former. This result resembles the increased bioaccessibility of rutin encapsulated by liposomes, rising from 9.8% to 19.7% [5], and lutein encapsulated in zein/soybean polysaccharide, rising from 16.2% to 32.1% [64]. The increased bioaccessibility of sesaminol in LYY microgel may be mainly related to the existence of sesaminol as nanoparticles and in an amorphous state [31]. However, the bioaccessibility of encapsulated sesaminol remained low, contributing to its poor water solubility [5].

### 3.10. Molecular Docking of Sesaminol with LYY and LYY Self-Assembly Structure

**Figure 8:**
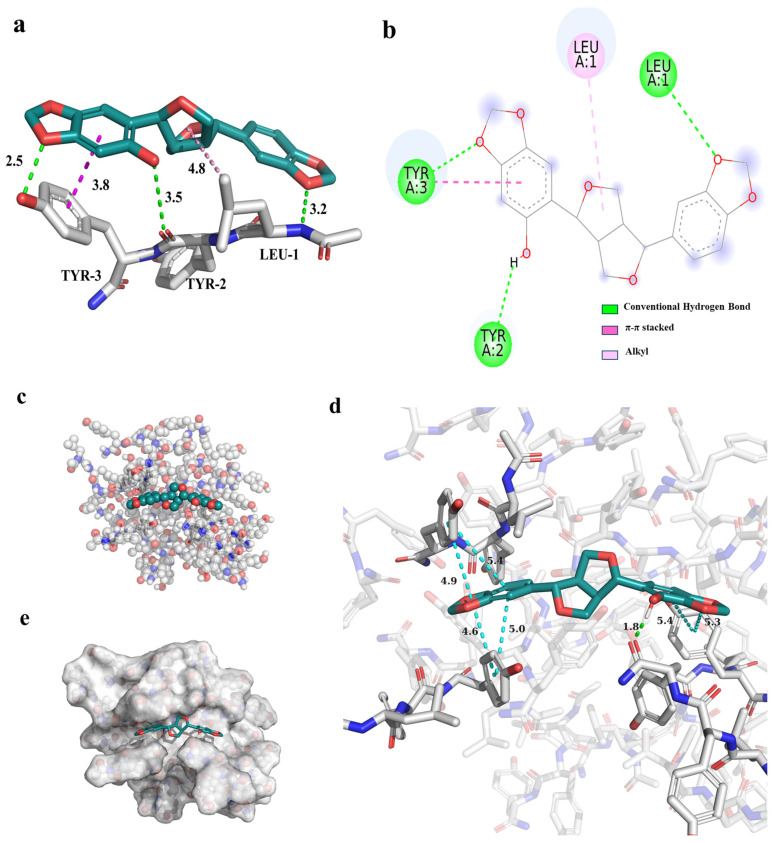
The interaction results between sesaminol and individual LYY peptides. (**a**) The 3D model (the dashed line represents the interaction, green represents hydrogen bonds, dark red represents π-π stacking, and light red represents alkyl interaction), (**b**) 2D model (the meaning represented by different colors are consistent with the 3D model). The docking results between sesaminol and LYY self-assembly structures. (**c**) Ball and stick model, (**d**) 3D model of interaction (green represents hydrogen bonds, and blue represents π-π stacking), (**e**) combined mode.

Molecular docking was performed to elucidate the interaction mechanism of sesaminol with free LYY. The interaction results are displayed in Figure 8a,b. The findings indicated that the sesaminol formed hydrogen bonds with L1 (LEUA:1), Y2 (TYRA:2), and Y3 (TYRA:3) of LYY, measuring 3.2 Å, 3.5 Å, and 2.5 Å in length. The side chain methyl of L1 underwent hydrophobic interactions (π-alkyl) with sesaminol, with a length of 4.8 Å. The Y3 of LYY involved π-π stacking with sesaminol, with a length of 3.8 Å. Furthermore, the free energy of −5.52 kcal/mol for the binding of sesaminol to LYY, with a binding energy of less than 0 kcal/mol (Table S1), indicates that the interaction between sesaminol and LYY is a beneficial, spontaneous, and exothermic process [65]. The primary interactions involved hydrogen bonding and van der Waals contact between sesaminol and individual LYY, which was −5.68 kcal/mol.

To further investigate the intermolecular interactions between sesaminol and LYY self-assembly molecules, the molecular docking was performed based on the stable structure obtained from the MD simulations of the LYY self-assembly microgel in Section 3.3.4 (Figure 8). The results indicated that sesaminol formed hydrogen bonds with the carbonyl group of the amide bond in the aggregate, spanning 1.8 Å. Furthermore, the fused rings at both ends of sesaminol formed π-π stacking interactions with the aromatic ring of the tyrosine side chain in the polypeptide, with a length of less than 5 Å. The results indicated that sesaminol can form hydrogen bonds and π-π stacking with both individual LYY and the LYY self-assembly structure. The binding model (Table S1) showed that the binding free energy between sesaminol and the LYY self-assembly structure was −8.66 kcal/mol, which is lower than the binding affinity of sesaminol with individual LYY, demonstrating a greater binding affinity towards the LYY self-assembly structure. The main interactions also involved hydrogen bonding and van der Waals contact between sesaminol and the LYY microgel, significantly contributing −7.56 kcal/mol. This provides strong evidence for the mechanism of interaction and the potential of binding interactions.

## 4. Conclusions

In this study, a self-assembled microgel was developed using the antioxidant peptide LYY. Furthermore, the microgel proved an effective encapsulation carrier for sesaminol, demonstrating a high encapsulation capacity. Compared to pure sesaminol, the composite microgel enabled the sustained release of sesaminol and improved its bioavailability and antioxidant properties. Furthermore, the analysis of the LYY self-assembly mechanism indicated that it primarily forms from *β*-folds and random coils, with the main interactive forces being hydrogen bonding and π-π stacking interactions. SEM and XRD results indicated that sesaminol was present in nanoparticles and amorphous structures within the encapsulation system. Molecular docking revealed that the primary interactions between sesaminol and individual LYY and its self-assembled structure were hydrogen bonding and π-π interactions. While the LYY peptide is demonstrated to be dual-functional as both an encapsulation carrier and an antioxidant, achieving effective encapsulation results, numerous translational challenges regarding industrial scalability, cost-effectiveness, and food safety persist. In future research, based on this work, we will strive to obtain functional peptides that can self-assemble from food and develop green solvent systems for constructing encapsulation systems for functional food components. Overall, this study provides molecular module support for constructing functional peptide encapsulation systems, improving the properties of sesaminol and promoting the development of the sesame processing industry. 

## Figures and Tables

**Table 1:** The physical and chemical properties of YY-derived peptides.

Entry	Sequence	Water Solubility (mg·mL^−1^)	Melting Point (°C)	Thermal Stability (°C)	IC_50_ of DPPH^+^ Scavenging Ability (μg/mL)	Cell Viability) (at 2.5 mmol of Peptide) %
1	LYY	0.65 ± 0.13	243.01	308.35	846.80	90.9 ± 2.1
2	RYY	44.70 ± 1.52	263.71	303.63	4970	85.3 ± 1.6
3	KYY	86.70 ± 2.17	292.43	338.96	74,000	91.8 ± 4.5
4	VYY	0.30 ± 0.08	280.67	304.26	84,000	87.6 ± 2.0
5	YLY	0.56 ± 0.09	260.00	313.30	6.186 × 10^6^	91.2 ± 3.6
6	YYL	0.84 ± 0.05	292.43	301.73	37.03	89.3 ± 3.8
7	IYY	0.44 ± 0.05	301.68	305.22	5600	91.8 ± 2.3
8	GYY	10.2 ± 0.95	289.82	303.35	5.695 × 10^6^	88.4 ± 2.9
9	AYY	2.60 ± 0.36	292.66	297.21	8116	86.45 ± 3.2
10	YY	16.92 ± 0.83	254.25	302.18	4279	91.2 ± 4.2

## Data Availability

The original contributions presented in this study are included in the article/Supplementary Material; further inquiries can be directed to the corresponding author.

## Supplementary Materials

The following supporting information can be downloaded at https://www.mdpi.com/article/10.3390/foods14060971/s1, Table S1: Docking fraction of sesaminol ligands and LYY self-assembly structures. Figure S1: (a) LC spectrum of LYY; (b) ^1^H NMR of LYY in DMSO-*d*_6_; (c) ESI-MS spectrum of LYY. Figure S2: (a) LC spectrum of IYY; (b) ^1^H NMR of IYY in DMSO-*d*_6_; (c) ESI-MS spectrum of IYY. Figure S3: (a) LC spectrum of VYY; (b) ^1^H NMR of VYY in DMSO-*d*_6_; (c) ESI-MS spectrum of VYY. Figure S4: (a) LC spectrum of AYY; (b) ^1^H NMR of AYY in DMSO-*d*_6_; (c) ESI-MS spectrum of AYY. Figure S5: (a) LC spectrum of GYY; (b) ^1^H NMR of GYY in DMSO-*d*_6_; (c) ESI-MS spectrum of GYY. Figure S6: (a) LC spectrum of KYY; (b) ^1^H NMR of KYY in DMSO-*d*_6_; (c) ESI-MS spectrum of KYY. Figure S7: (a) LC spectrum of RYY; (b) ^1^H NMR of RYY in DMSO-*d*_6_; (c) ESI-MS spectrum of RYY. Figure S8: (a) LC spectrum of YLY; (b) ^1^H NMR of YLY in DMSO-*d*_6_; (c) ESI-MS spectrum of YLY. Figure S9: (a) LC spectrum of YYL; (b) ^1^H NMR of YYL in DMSO-*d*_6_; (c) ESI-MS spectrum of YYL. Figure S10: Molecular structures (a) and TEM images (b–j) of YY-derived tripeptide self-assemblies in HFIP–water binary solvents (*v*:*v* = 3:2). Figure S11. The antioxidant activity of LYY-based microgel. Figure S12: Intermolecular interactions of LYY: (a) hydrogen bond and (b) π-π stacking. Figure S13. (a) HPLC Chromatograms of simulated digestion of the sesaminol-LYY microgels. (b) HPLC Chromatograms of LYY standards. (c) HPLC Chromatograms of sesaminol standards.
