# Supplementary material for: Design and Characterization of Peptide-Based Self-Assembling Microgel for Encapsulation of Sesaminol
Source: Foods. 2025 Mar 12;14(6):971. doi: 10.3390/foods14060971 (PMC11941722; doi:10.3390/foods14060971)
Supplement: Supplementary file 1 [file foods-14-00971-s001.zip › foods-3510051-supplementary.docx]

**Supplementary Material**

**Design and Characterization Peptide-based Self-Assembling Microgel for Encapsulation of Sesaminol**

Jinhong Gao^a^, Heng Du^a^, Zhenhong Zhang^b^, Qunpeng Duan^c^, Libo Yuan^b^, Bingchao Duan^d^, Hongyan Yang^d^, Pengcheng Meng^a^, Kui Lu^a, d*^

^a^ College of Food Science and Engineering, Henan University of Technology, Zhengzhou 450001, China.

^b^ School of Chemistry and Chemical Engineering, Henan University of Technology, Zhengzhou 450001, China.

^c^ School of Chemistry and Printing-Dyeing Engineering, Henan University of Engineering, Zhengzhou 450007, China.

^d^ School of Food Science and Chemical Engineering, Zhengzhou University of Technology, Zhengzhou 450044, China.

**First author:**

**Jinhong Gao**, E-mail: [jinhonggao@126.com](mailto:jinhonggao@126.com); orcid.org/ 0000-0002-8536-6807.

**^*^Corresponding author:**

**Kui Lu**, E-mail: [lukui126@126.com](mailto:lukui126@126.com); orcid.org/0000-0001-9730-485X.

1. **Characterization and properties of YY-derived peptides**

*The method of HPLC*

The purified products were detected according to semi-preparative liquid chromatography (Agilent 1260, Agilent, CA, US) with a UV detector and an Agilent Zorbax ODS (9.4 mm × 250 mm, 5 μm). The temperature of the column oven was 30 ℃. The injection volume was 10 μL. The mobile phase was water (A) and acetonitrile with 0.1% trifluoroacetic acid (B) with the following system: 1 mL/min, 60% B, 20 min.

*The melting point and thermal stability*

All peptides' melting points and thermal stability were determined using simultaneous thermal analysis TG-DSC (Mettler TGA/DSC1) (Table S1). 5 mg of the sample was measured and put into a crucible. Under an N_2_ atmosphere, the temperature was increased from room temperature to 600 ℃ at a rate of 10 ℃/min. Changes in the sample's mass and heat flow rate were documented.

*The water solubility*

100 mg of the sample was weighed accurately and placed in a 50 mL centrifuge tube. Then, 2 mL of deionized water was added and mixed well using a vortex. After that, the tube was put in a constant temperature shaker at 25 ℃ and shaken at 100 r/min for 24 h. Next, the sample was transferred to a 5 mL centrifuge tube and centrifuged at 10000 r/min for 30 min. After centrifugation, the supernatant was transferred to a 50 mL centrifuge tube and frozen dry for 48 h. Finally, the sample was weighed, and the weight was used to calculate the solubility of the samples.

*Cell Viability Assay*

To assess the effect of different concentrations of LYY on cell viability, HeLa and HEK-293 cells were used as target cells, and an MTT assay was performed. LYY power was dissolved in a 5% (v/v) DMSO phosphate buffer solution and then diluted to different concentrations. Next, the solution was added to a 96-well plate with around 5000 cells/well. Following 24 h of incubation, the absorbance value was measured at 595 nm using an enzyme-linked immunosorbent assay (ELISA) reader. A 5% (v/v) DMSO phosphate buffer solution was used as a control. In addition, the same volume of peptide solution was added as a blank to the cell-free culture medium to calculate the cell viability value.

*Antioxidant ability*

The DPPH free radical scavenging ability of peptides, sesaminol, and tea polyphenols was measured. 100 μL samples of various concentrations were mixed with DPPH in 80% ethanol (0.2 mmol/L) in a 96-well microplate. The mixture was incubated in the dark at room temperature for 30 minutes. The absorbance (Abs) was measured using an Infinite M Nano absorbance plate reader (Tecan Group Ltd., Zürich, Switzerland) at 517 nm. The ability of the DPPH radical scavenging was obtained using equation (1). The IC_50_ values of each sample were calculated using GraphPad Prism 5.

$\text{DPPH}_{\text{scavenging ability}}\text{=}\left( \text{1-}\frac{\text{A}_{\text{sample}}\text{-}\text{A}_{\text{sample background}}}{\text{A}_{\text{control}}} \right)\text{×100\%}$ (1)

A_sample_ represented the Abs of the sample and DPPH, A_sample background_ represented the Abs of the sample background (sample and ethanol instead of DPPH), and A_control_ represented the Abs of the control (DPPH and 100 μL of ethanol instead of the sample).

**Table S1**. Docking fraction of sesaminol ligands with individual LYY peptides and LYY self-assembly structures

| **Number** | **Energy** | **Individual LYY** | **LYY self-assembly structures** |
| --- | --- | --- | --- |
| **1** | **Estimated free energy of binding (ΔG, kcal/mol)** | -5.52 | -8.66 |
| **2** | **Estimated dissociation constant (Kd, μM)** | 90.40 | 0.45 |
| **3** | **Final intermolecular energy (kcal/mol)** | -5.86 | -9.19 |
|  | Hydrogen bond+ van der Waals Energy (kcal/mol) | -5.78 | -7.56 |
|  | Electrostatic Energy (kcal/mol) | -0.08 | -1.63 |
| **4** | **Final total internal energy (kcal/mol)** | -1.14 | -1.17 |
| **5** | **Torsional free energy (kcal/mol)** | 0.89 | 0.89 |
| **6** | **Unbound system energy (kcal/mol)** | -0.59 | -0.81 |

Note：The formula of binding free energy calculation: ΔG = (3)+(4)+(5)-(6);

The formula of Kd: Kd = ln (ΔG/RT), R is constant 8.314 J/mol·K, T is temperature 298.15 K.

1. **Identification of derived peptides**

***LYY***

Purity: 99.94 %. ^1^H NMR (400 MHz, DMSO-*d_6_*) δ (ppm) =9.40-8.84 (br, 2H), 7.98 (d, *J* = 8 Hz, 1H), 7.88-7.83 (m, 2H), 7.26 (s, 1H), 7.10 (s, 1H), 7.00-6.94 (m, 4H), 6.64-6.58 (m, 4H), 4.34-4.29 (m, 2H), 4.24-4.19 (m, 1H), 2.88-2.81 (m, 2H), 2.74-2.62 (m, 2H), 1.81 (s, 1H), 1.54-1.44 (m, 1H), 1.32 (t, *J* = 7.4 Hz, 2H), 0.85-0.79 (m, 6H). MS: calcd M=498.52, obsd [M+H] ^+^ =499.25, obsd [2M+H] ^+^=997.00, obsd [2M+Na] ^+^=1019.00.


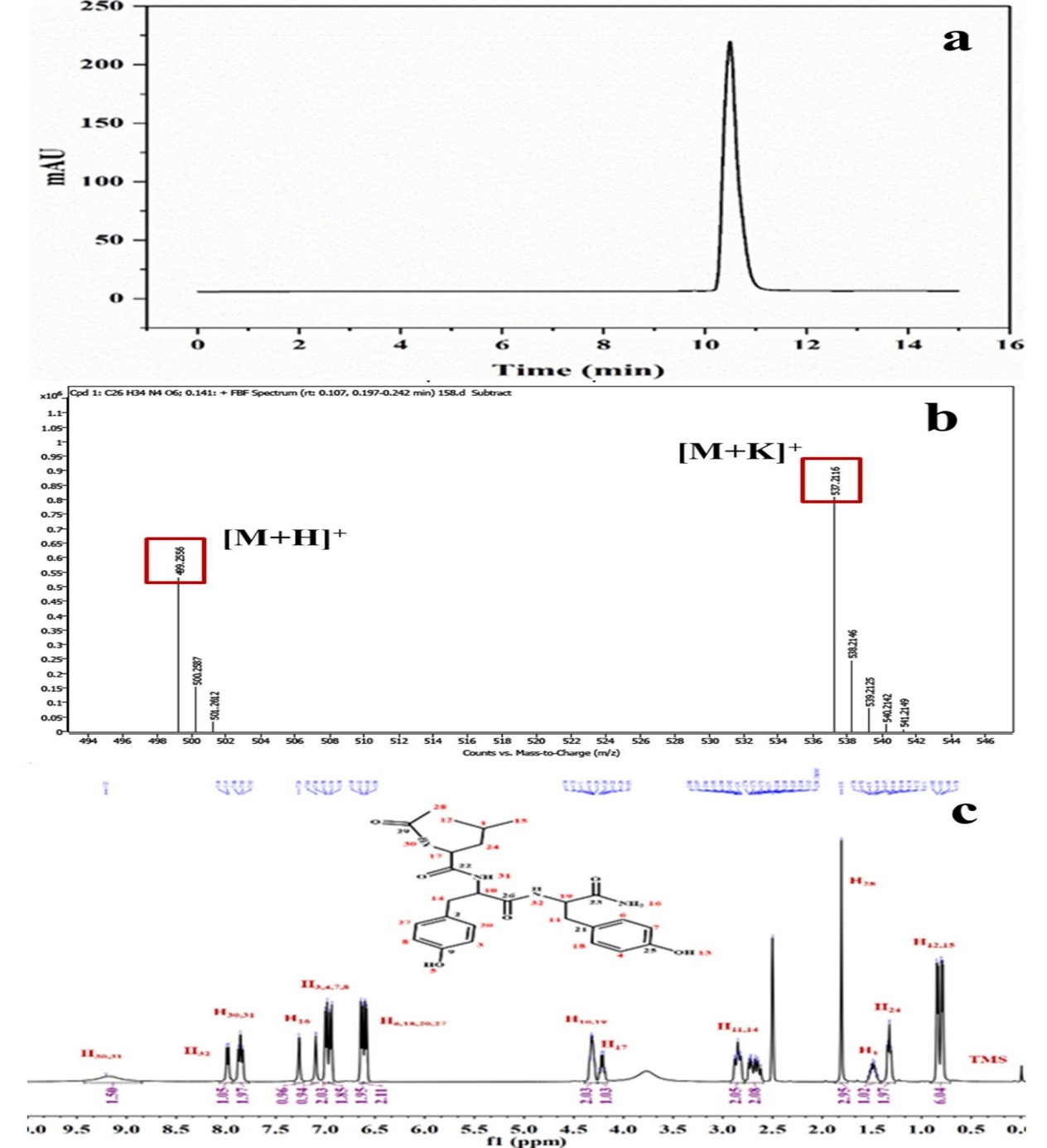


**Figure S1.** **(**a) LC spectrum of LYY); (b) ^1^H NMR of LYY in DMSO-*d_6_*; (c) ESI-MS spectrum of LYY.

***IYY***

Purity: 97.65 %. ^1^H NMR (400 MHz, DMSO-*d_6_*) δ (ppm) = 9.14 (s, 1H), 9.13 (s, 1H), 7.92 (d, *J* = 8 Hz, 1H), 7.83 (d, *J* =8 Hz,1H), 7.77 (d, *J* =8 Hz, 1H), 7.20 (s, 1H), 7.04 (s, 1H), 6.98 (d, *J* =8 Hz, 2H), 6.95 (d, *J* =8 Hz, 2H), 6.63 (d, J =8 Hz, 2H), 6.58 (d, J =8 Hz, 2H), 4.39-4.30 (m, 2H), 4.09 (t, *J* =8 Hz, 1H), 2.88-2.80 (m, 2H), 2.75-2.69 (m, 1H), 2.66-2.60 (m, 1H), 1.84 (s, 3H),1.68-1.57 (m,1H), 1.34-1.26 (m, 1H), 0.96-1.04 (m, 1H), 0.76 (t, *J* =7.4 Hz, 3H ),0.70 (d, *J* =6.8 Hz, 3H). MS: calcd M=498.51, obsd [M+H] ^+^ =498.92.


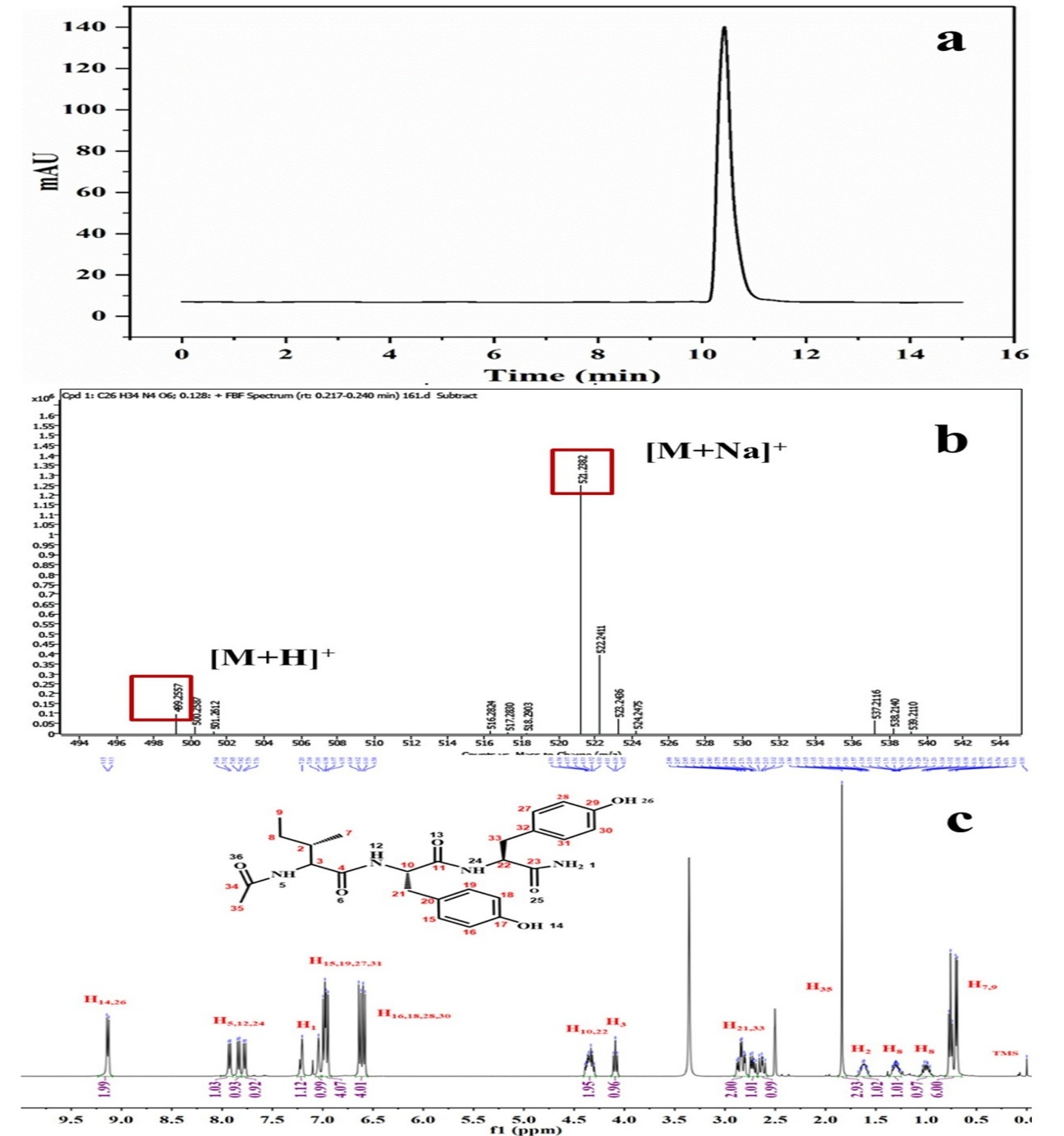


**Figure S2.** **(**a) LC spectrum of IYY; (b) ^1^H NMR of IYY in DMSO-*d_6_*; (c) ESI-MS spectrum of IYY.

***VYY***

Purity: 99.45 %. ^1^H NMR (400 MHz, DMSO-*d_6_*) δ (ppm) = 9.15-9.14 (m, 2H), 7.93 (d, *J*=8 Hz, 2H), 7.82-7.77(m, 2H), 7.2 (s, 1H), 7.04 (s, 1H), 7.0-6.96 (m, 4H), 6.58-6.64(m, 4H), 4.38-4.30 (m, 2H), 4.08 (t, *J* =7.8 Hz, 1H), 2.88-2.80 (m, 2H), 2.75-2.70 (m,1H), 2.66-2.60 (m, 1H), 1.91-1.86(m, 1H), 1.85,(s,3H), 0.74 (d, *J* = 6.8 Hz, 6H). MS: calcd M=484.49, obsd [M+H] ^+^ =485.00, obsd [M+Na] ^+^ =507.28, obsd [2M+H] ^+^=968.67, obsd [2M+Na] ^+^=990.67.


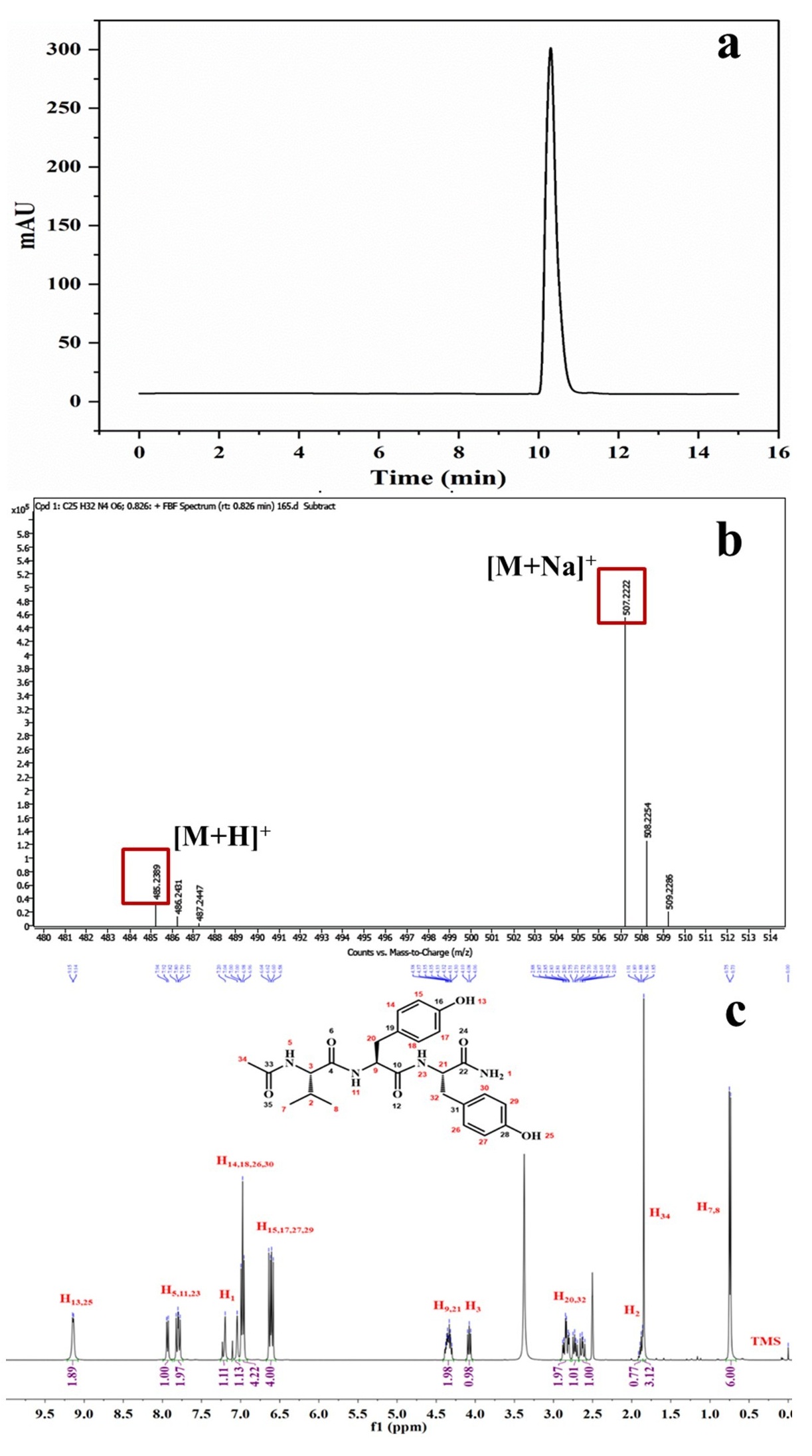


**Figure S3.** **(**a) LC spectrum of VYY; (b) ^1^H NMR of VYY in DMSO-*d_6_*; (c) ESI-MS spectrum of VYY.

***AYY***

Purity: 99.94 %. ^1^H NMR (400 MHz, DMSO-*d_6_*) δ (ppm) =9.15 (s, 1H), 9.13 (s,1H), 8.02 (d, *J* = 8.4 Hz,1H), 7.85 (d, *J* =8.8 Hz, 1H), 7.81 (d, *J* =8 Hz, 1H), 7.23 (s, 1H), 7.07(s, 1H), 7.01-6.94 (m, 4H), 6.66-6.90 (d, 4H), 4.35-4.28 (m, 2H), 4.17-4.24 (m, 1H), 2.91-2.82 (m, 2H), 2.75-2.69 (m, 1H), 2.66-2.63 (m, 1H), 1.82 (s, 3H), 1.68-1.57 (m, 1H), 1.34-1.26 (m, 1H), 1.04-0.96 (m, 1H). MS: calcd M=456.44, obsd [M+H] ^+^ =457.17, obsd [M+Na] ^+^ =479.42, obsd [2M+H] ^+^=912.75, obsd [2M+Na] ^+^=935.00.


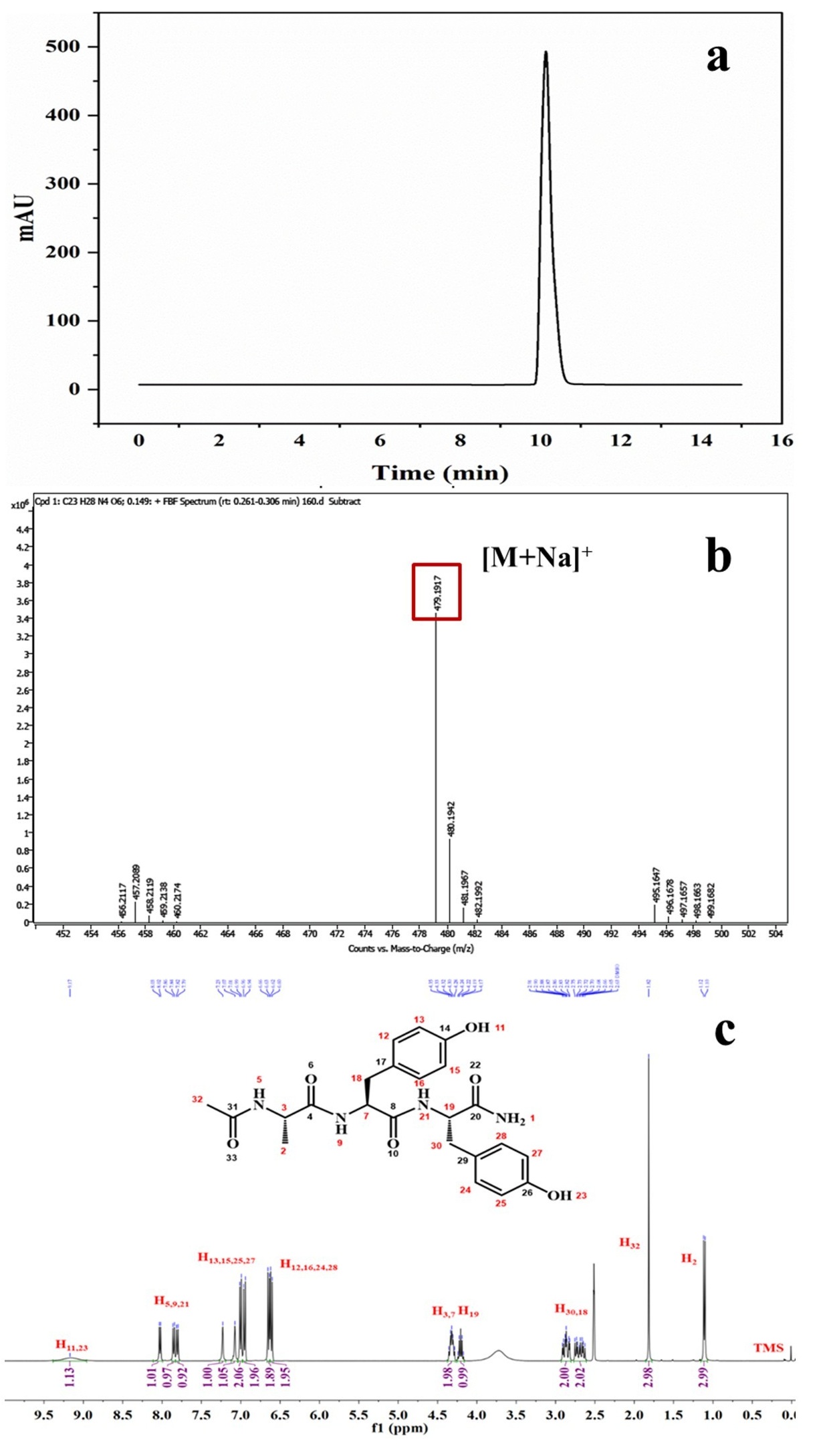


**Figure S4.** **(**a) LC spectrum of AYY; (b) ^1^H NMR of AYY in DMSO-*d_6_*; (c) ESI-MS spectrum of AYY.

***GYY***

Purity: 99.85 %. ^1^H NMR (400 MHz, DMSO-*d_6_*) δ (ppm) = 9.24-9.08 (br, 2H), 8.11 (t, *J* =6.8 Hz, 1H), 7.98 (d, J = 8Hz, 1H), 7.93 (d, J = 8.4 Hz, 1H), 7.19 (s, 1H), 7.07 (s, 1H), 7.02-6.95 (m, 4H), 6.66-6.60 (m, 4H), 4.37-4.28 (m, 2H), 3.72-3.66 (m, 1H), 3.58-3.52 (m, 1H), 2.81-2.92 (m, 2H), 2.74-2.69 (m, 1H), 2.62-2.56 (m, 1H), 1.80 (s, 3H). MS: calcd M=442.47, obsd [M+Na] ^+^ = 465.00.


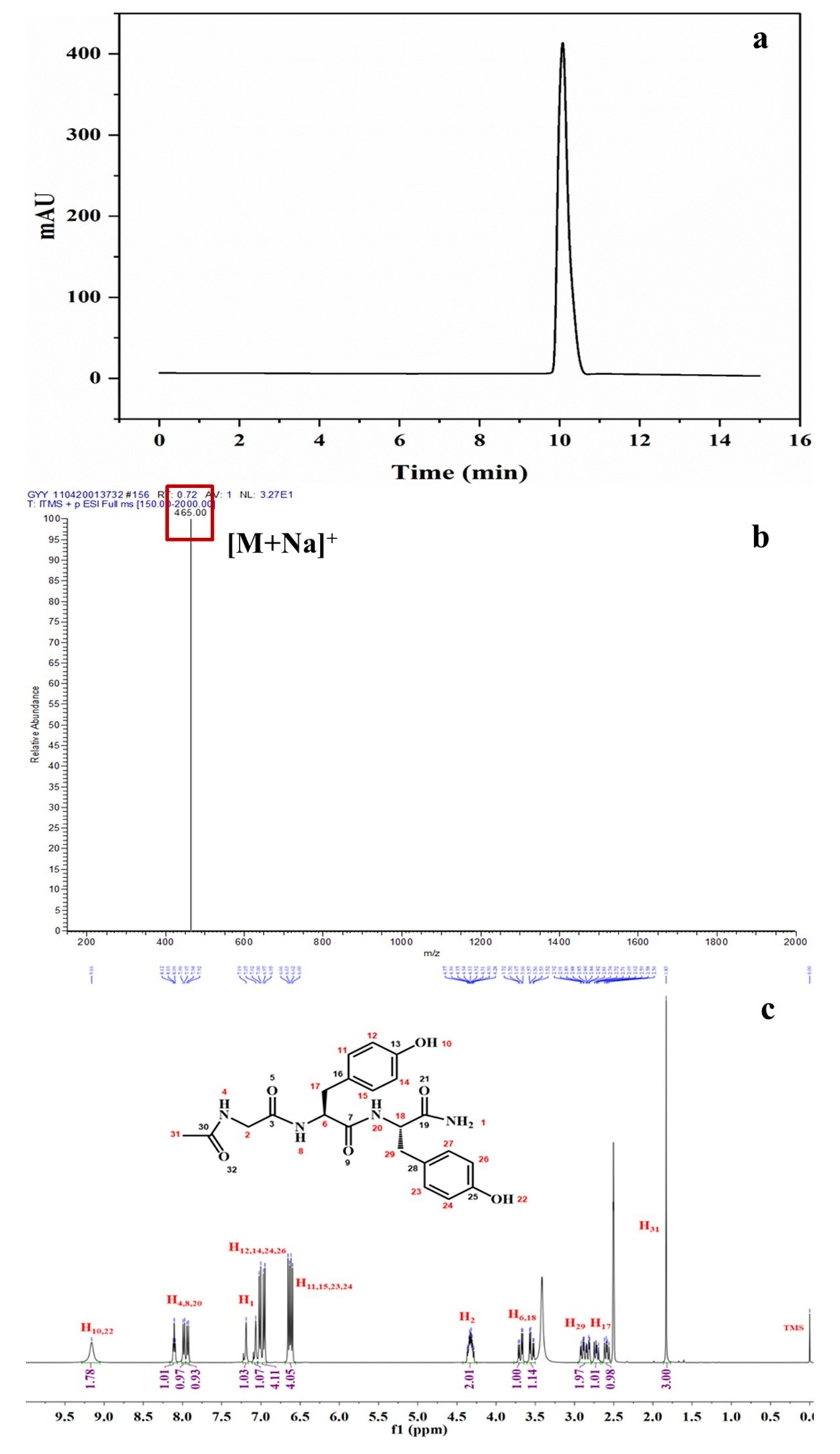


**Figure S5.** (a) LC spectrum of GYY; (b) 1H NMR of GYY in DMSO-d6; (c) ESI-MS spectrum of GYY.

***KYY***

Purity: 99.43 %. ^1^H NMR (500 MHz, DMSO-*d_6_*) δ (ppm) = 9.33-9.09 (br, 2H), 7.99 (d, *J*=8 Hz, 2H), 7.90 (d, *J*=8 Hz, 2H), 7.83 (d, *J*=8 Hz, 2H), 7.73 (s, 2H), 7.30 (s, 1H), 7.07 (s, 1H), 7.01-6.94 (m, 4H), 6.66-6.60 (m, 4H), 4.36-4.30 (m, 2H), 4.20-4.14 (m, 1H), 2.90-2.82 (m, 2H), 2.75-2.61 (m, 4H), 1.83 (s, 3H), 1.54-1.45 (m, 4H), 1.29-1.13 (m, 2H). MS: calcd M=513.53, obsd [M+H] ^+^ =514.42, obsd [2M+H] ^+^=1027.08.


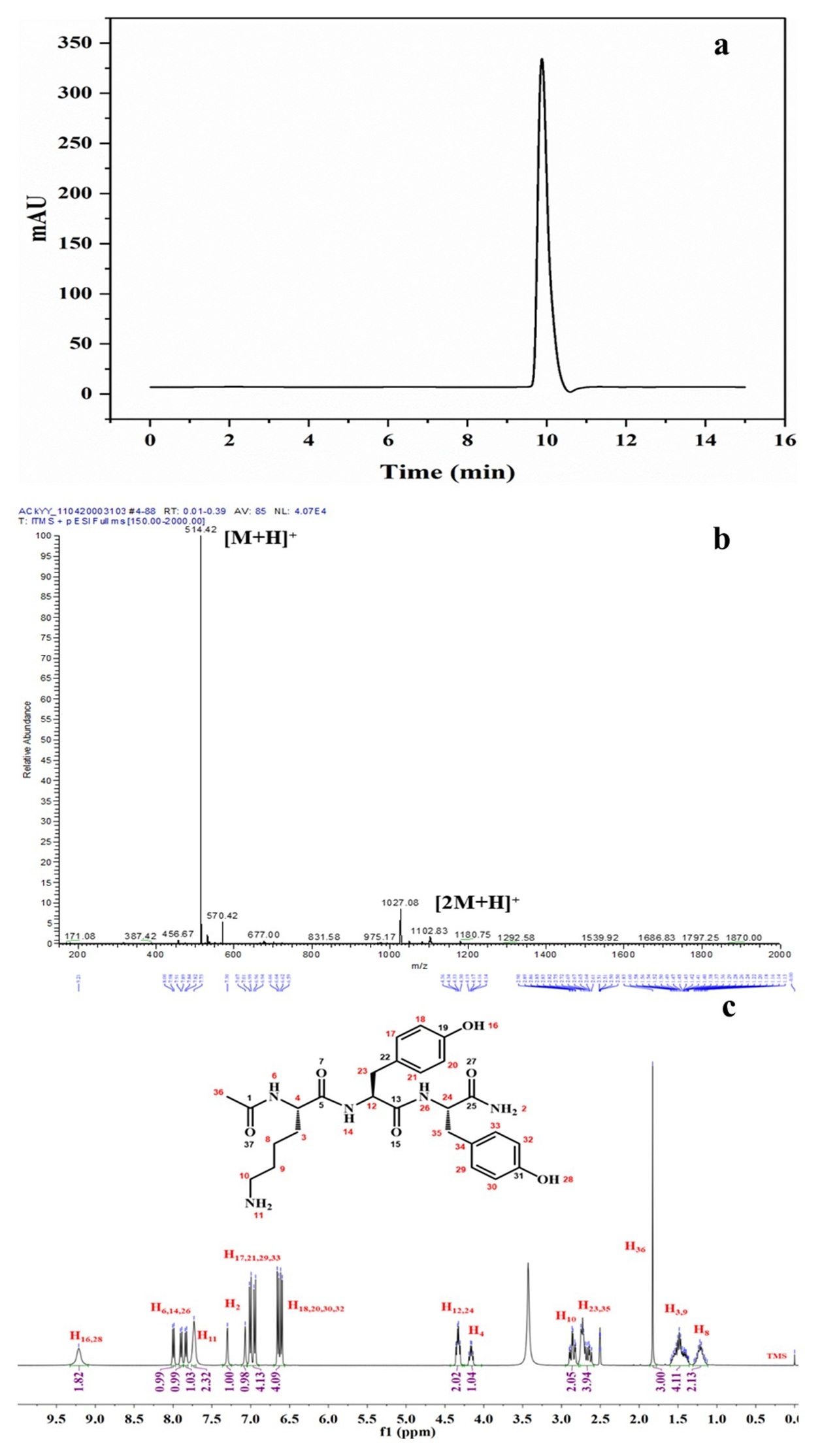


**Figure S6.** **(**a) LC spectrum of KYY; (b) ^1^H NMR of KYY in DMSO-*d_6_*; (c) ESI-MS spectrum of KYY.

***RYY***

Purity: 99.85 %. ^1^H NMR (400 MHz, DMSO-*d*_6_) δ (ppm) = 9.33-9.09 (br, 2H), 8.03 (d, *J* = 8 Hz,1H), 7.91-7.89 (m, 2H), 7.54-7.03 (m, 6H), 7.03 - 6.94 (m, 4H), 6.66-6.59 (m, 4H), 4.37-4.31 (m, 2H), 4.25-4.20 (m, 1H), 3.13-3.01 (m, 2H), 2.90-2.82 (m, 2H), 2.75-2.62 (m, 2H), 1.84 (s, 3H), 1.63-1.54 (m, 1H), 1.44-1.37 (m, 3H). MS: calcd M=541.55, obsd [M+H] ^+^ =542.50.


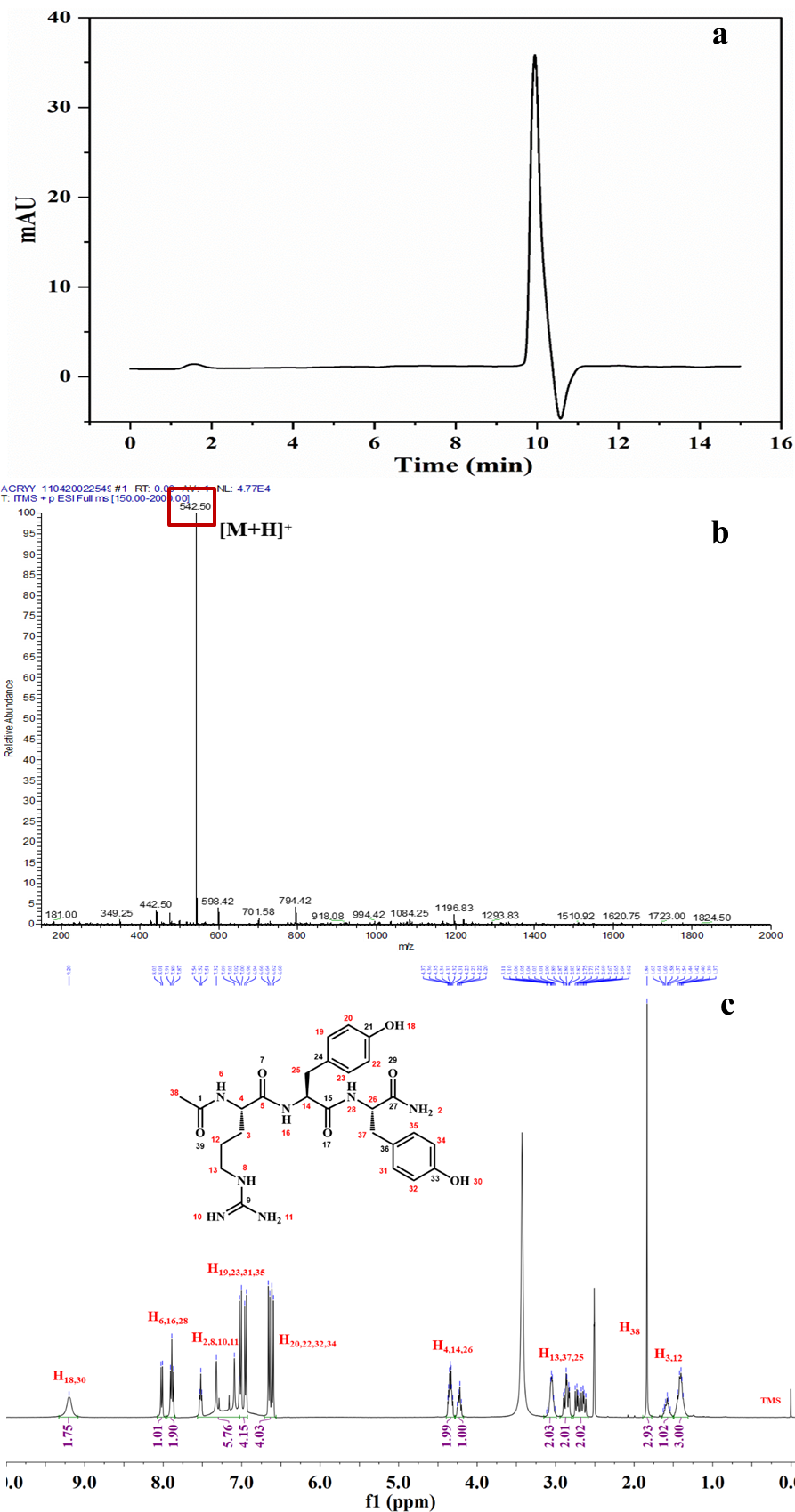


**Figure S7.** **(**a) LC spectrum of RYY; (b) ^1^H NMR of RYY in DMSO-*d_6_*; (c) ESI-MS spectrum of RYY.

***YLY***

Purity:99.98 %. ^1^H NMR (400 MHz, DMSO-*d*_6_) δ (ppm) = 9.22-8.81 (dr, 2H), 8.07-9.02 (m, 2H), 7.65 (d, 1H), 7.27 (s, 1H), 7.05 (s, 1H), 7.04-6.96 (m, 4H), 6.64-6.61 (m, 4H), 4.42-4.30 (m, 2H), 4.21-4.15 (m, 1H), 2.91-2.83 (m, 2H), 2.75-2.69 (m, 1H), 2.63-2.57 (m, 1H), 1.75 (s, 3H), 1.57-1.47 (m, 1H), 1.43-1.35 (m, 2H), 0.87- 0.81 (m, 6H). MS: calcd M=498.52, obsd [M+H] ^+^ = 498.75.


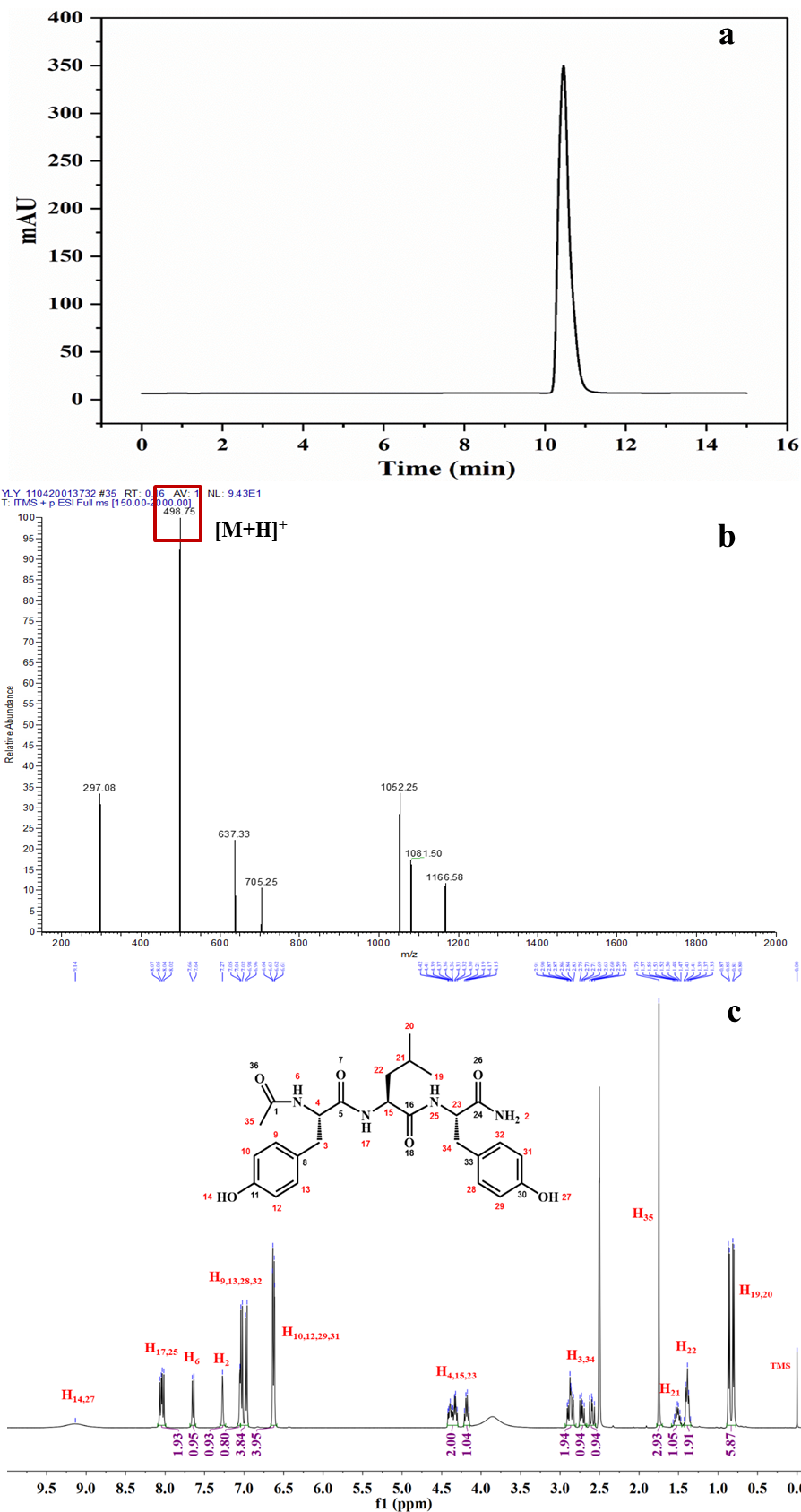


**Figure S8.** **(**a) LC spectrum of YLY; (b) ^1^H NMR of YLY in DMSO-*d_6_*; (c) ESI-MS spectrum of YLY.

***YYL***

Purity: 99.98 %. ^1^H NMR (400 MHz, DMSO-*d*_6_) δ (ppm) =9.96-8.43(br, 2H), 8.01-7.98 (m, 2H), 7.83 (d, *J*=8 Hz, 1H), 7.13 (s, 1H), 7.03 (s, 1H), 7.01-6.95 (m, 4H), 6.64-6.60 (m, 4H), 4.42-4.31 (m, 2H), 4.24-4.18 (m, 1H), 2.95-2.90 (m, 1H), 2.82-2.78 (m, 1H), 2.74-2.69 (m, 1H), 2.58-2.52 (m, 1H), 1.73 (s, 3H), 1.62-1.50 (m, 1H), 1.48-1.43 (m, 1H),0.89-0.83 (m, 6H). MS: calcd M=498.52, obsd [M+H] ^+^ =499.17, obsd [2M+H] ^+^=997.00, obsd [2M+Na] ^+^=1018.92.


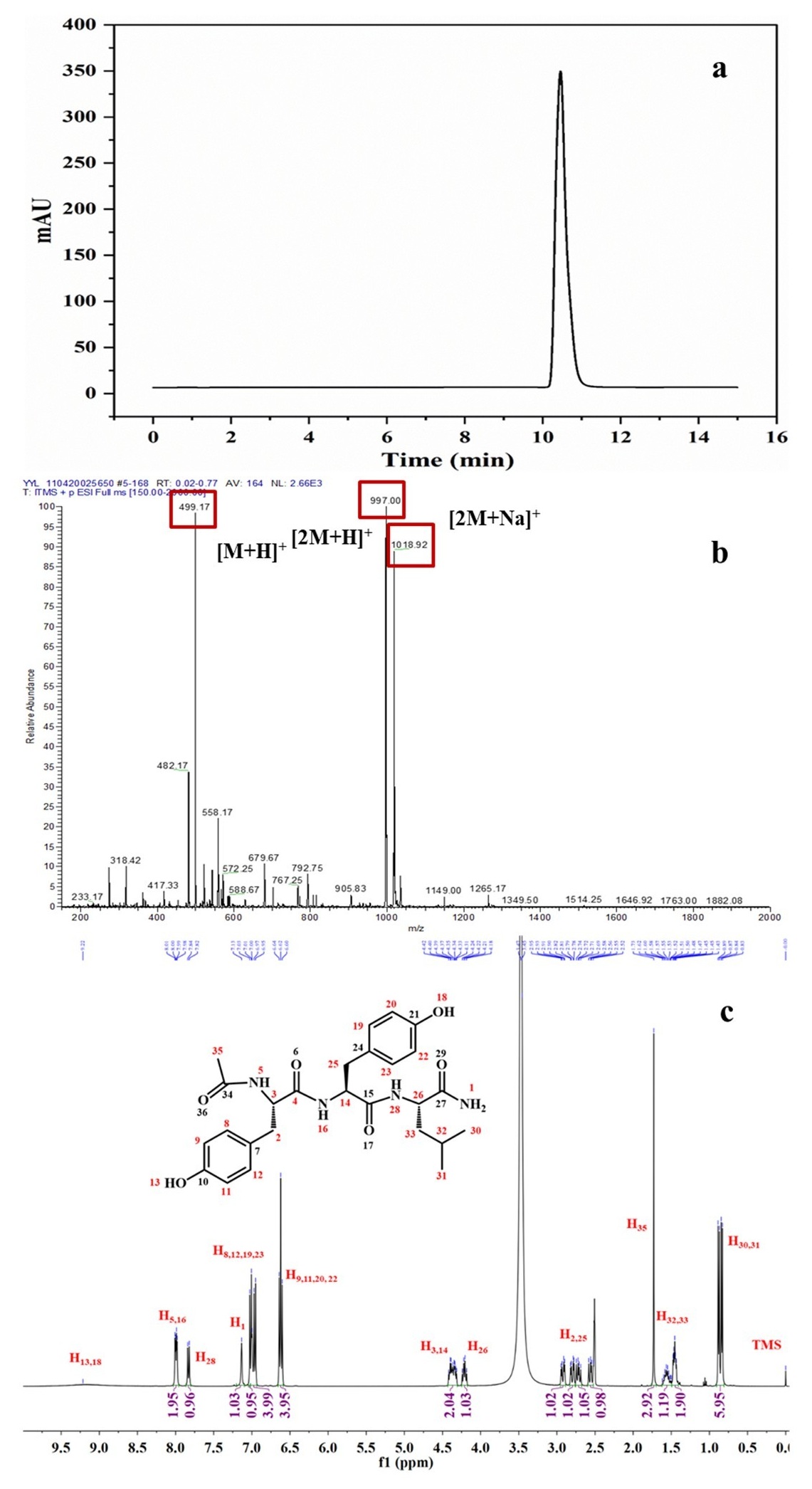


**Figure S9.** **(**a) LC spectrum of YYL; (b) ^1^H NMR of YYL in DMSO-*d_6_*; (c) ESI-MS spectrum of YYL.

1. Molecular structures and microstructure characteristics of YY-derived tripeptide self-assemblies


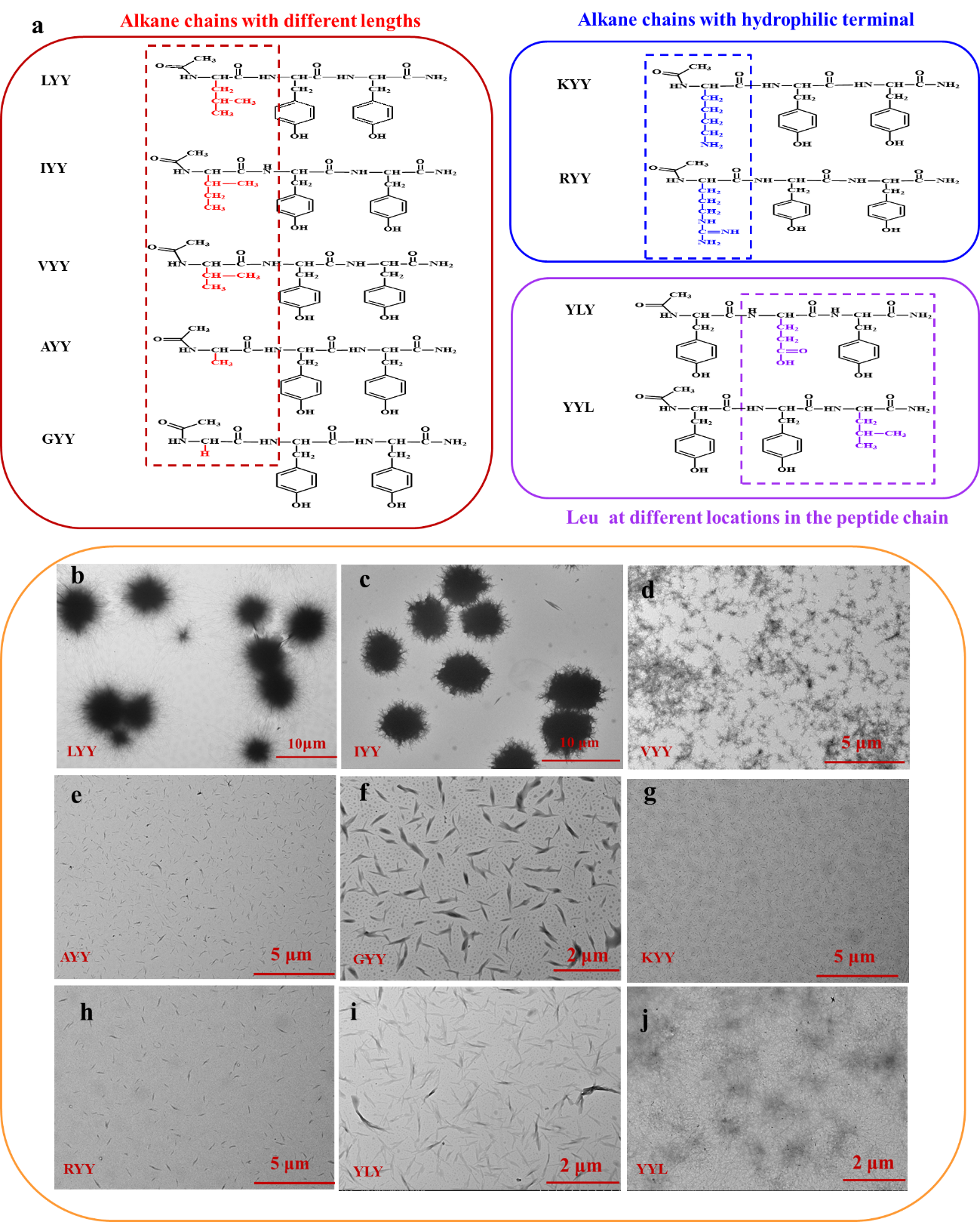


**Figure S10.** Molecular structures (a) and TEM images (b-j) of YY-derived tripeptide self-assemblies in HFIP-water binary solvents (v: v= 3:2).

1. **The antioxidant activity of LYY-based microgel**


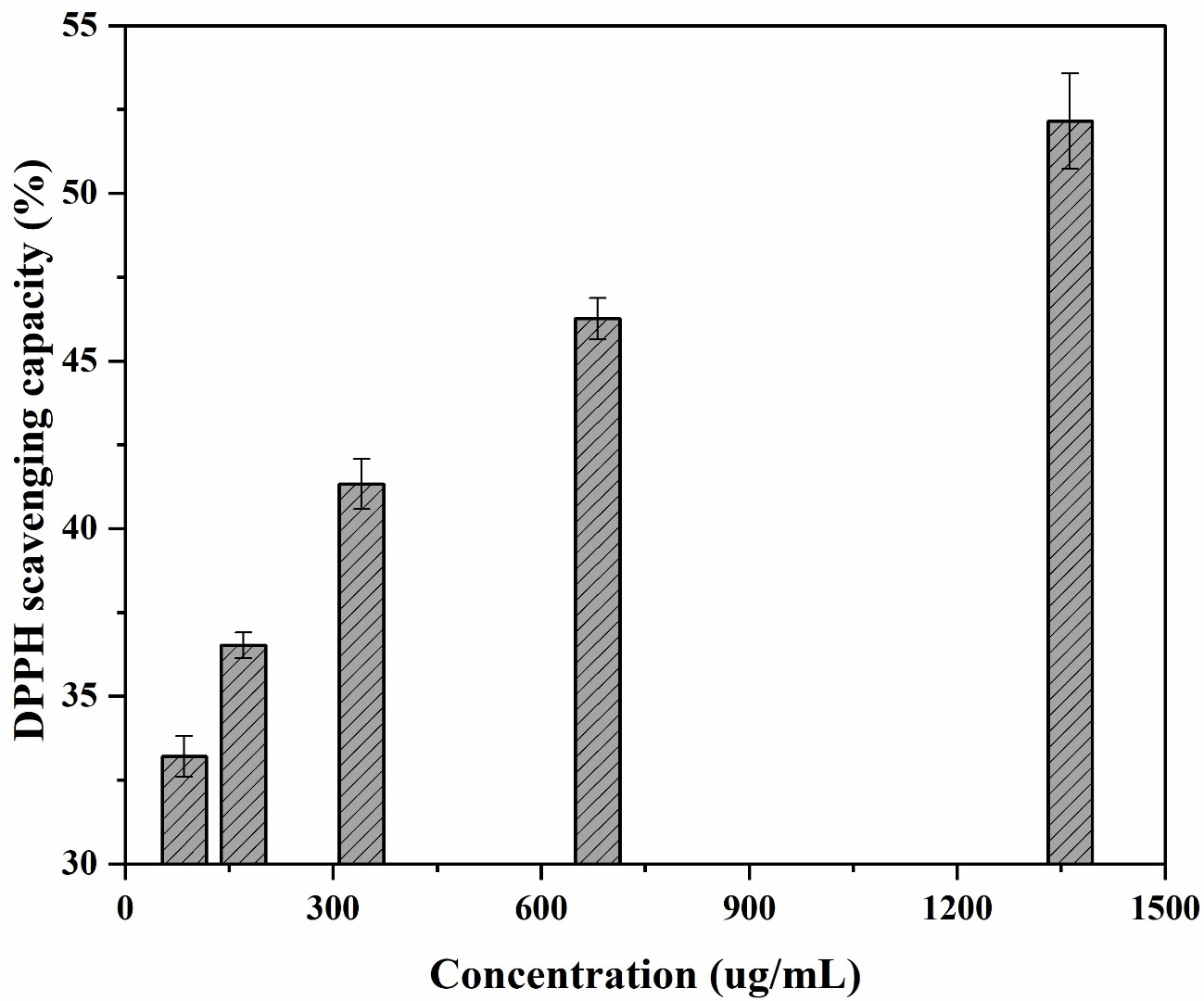


**Figure S11. The antioxidant activity of LYY-based microgel**

1. **The molecular mechanism of LYY self-assembly**


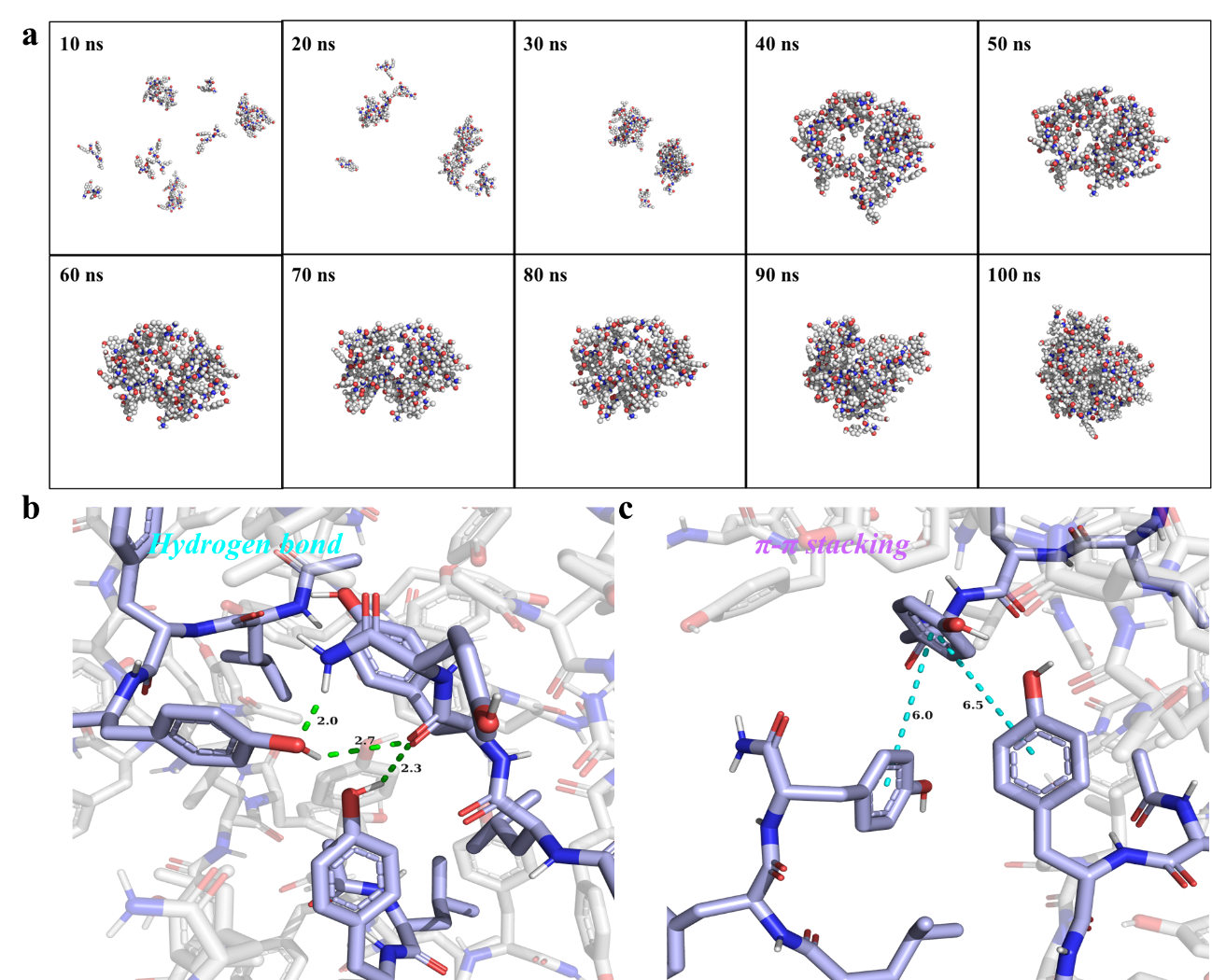


**Figure S12.** (a) the structural changes in MD simulation of the peptide self-assembly process (10-100 ns). Intermolecular interactions of LYY. (b) Hydrogen bond, (c) π-π stacking.


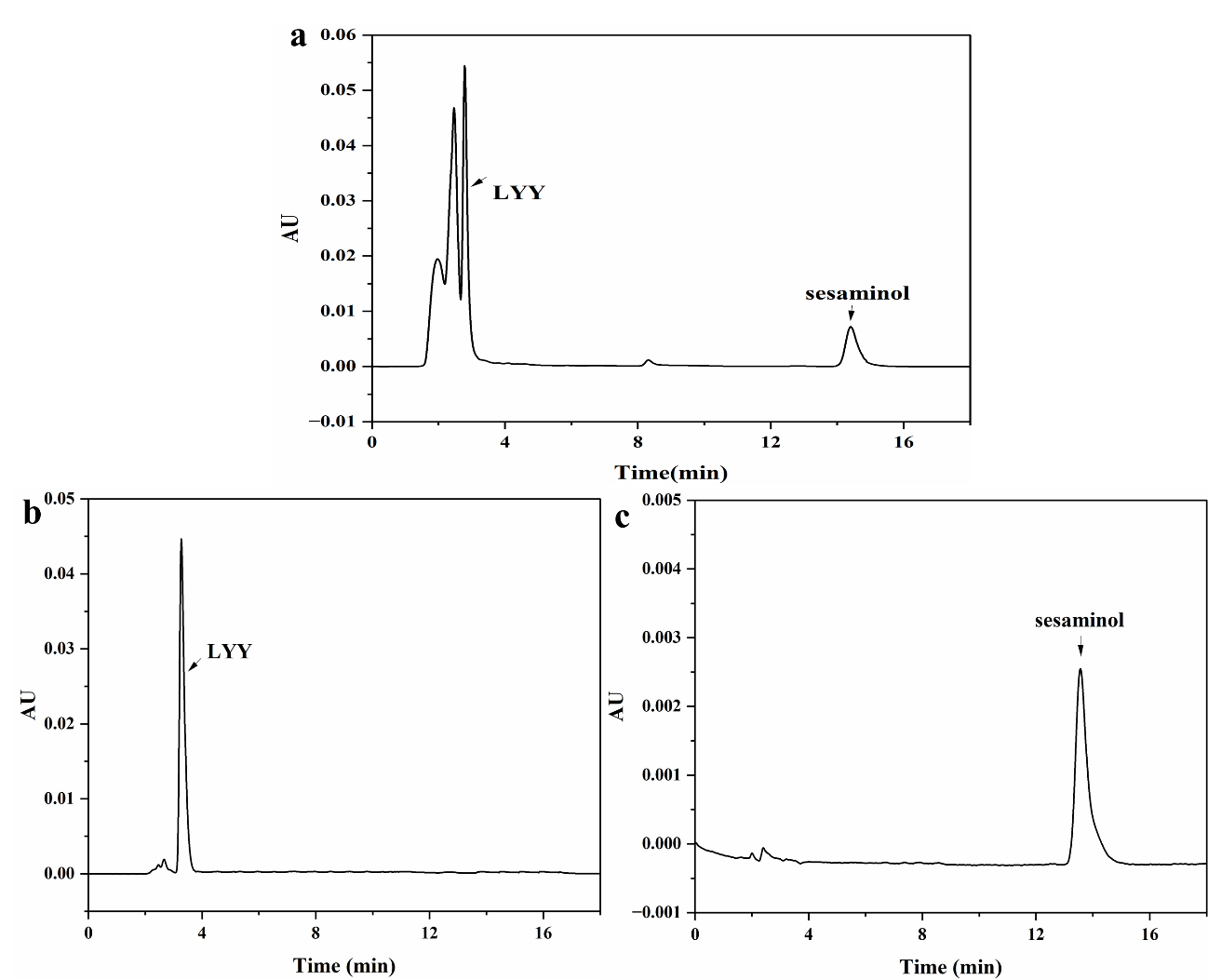


**Figure S13**. (a) HPLC Chromatograms of simulated digestion of the sesaminol-LYY microgels. (b) HPLC Chromatograms of LYY standards. (c) HPLC Chromatograms of sesaminol standards.
